# Three-Dimensional Representation of Motor Space in the Mouse Superior Colliculus

**DOI:** 10.1016/j.cub.2018.04.021

**Published:** 2018-06-04

**Authors:** Jonathan J. Wilson, Nicolas Alexandre, Caterina Trentin, Marco Tripodi

**Affiliations:** 1 MRC Laboratory of Molecular Biology, Cambridge, UK

**Keywords:** superior colliculus, 3D, motor control, space encoding

## Abstract

From the act of exploring an environment to that of grasping a cup of tea, animals must put in register their motor acts with their surrounding space. In the motor domain, this is likely to be defined by a register of three-dimensional (3D) displacement vectors, whose recruitment allows motion in the direction of a target. One such spatially targeted action is seen in the head reorientation behavior of mice, yet the neural mechanisms underlying these 3D behaviors remain unknown. Here, by developing a head-mounted inertial sensor for studying 3D head rotations and combining it with electrophysiological recordings, we show that neurons in the mouse superior colliculus are either individually or conjunctively tuned to the three Eulerian components of head rotation. The average displacement vectors associated with motor-tuned colliculus neurons remain stable over time and are unaffected by changes in firing rate or the duration of spike trains. Finally, we show that the motor tuning of collicular neurons is largely independent from visual or landmark cues. By describing the 3D nature of motor tuning in the superior colliculus, we contribute to long-standing debate on the dimensionality of collicular motor decoding; furthermore, by providing an experimental paradigm for the study of the metric of motor tuning in mice, this study also paves the way to the genetic dissection of the circuits underlying spatially targeted motion.

## Highlights

• Development of inertial sensor system for monitoring 3D head movements in real time
• Neurons in the superior colliculus code for the full dimensionality of head rotations
• Firing rate correlates with velocity, but not head displacement angle
• The spatial tuning of collicular units is largely independent of visual or landmark cues

## Graphical Abstract

**Figure:**
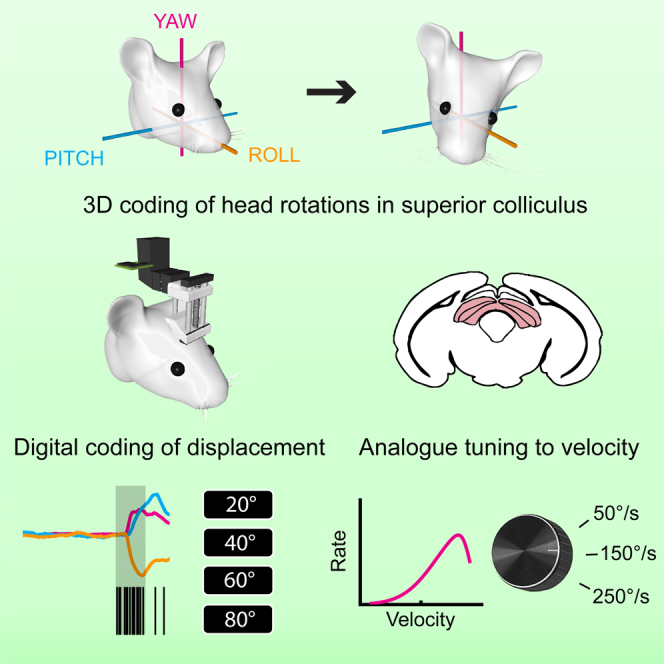

## Teaser

Enacting spatially tuned movements requires a representational map of 3D motion vectors. Wilson et al. show that superior colliculus (SC) neurons are tuned to the full dimensionality of head rotations. Individual SC neurons code for preferred displacement angles, whereas their firing rates are further tuned to the angular velocity of movement.

## Introduction

In recent years, we came to understand how animals encode spatial information in the context of navigation, how perceptual spatial maps are constructed, and how the metric of space is encoded [1, 2, 3]. More recently, the conceptual need of extending these maps in the three dimensions has emerged and the coding principles have begun to be unraveled [4, 5, 6]. Similarly, spatially tuned actions may rely on a map of surrounding space, egocentric in nature and whose implementation becomes overt in the motor domain with the decoding of appropriate displacement vectors.

The neural mechanisms involved in the decoding of such displacement vectors have been prominently characterized in primates and cats [7, 8, 9]. In particular, work on the role of the superior colliculus (SC) in the control of saccades [10, 11, 12] and gaze shifts (combined head and eye movements) [13, 14, 15, 16] has been instrumental for our understanding of the neural coding strategies underlying spatially tuned movements. However, the use of these less genetically amenable model systems has limited the functional dissection of the networks in the SC that are involved in guiding spatially tuned movements. Translating this line of studies into a genetically amenable model might open a new frontier into the investigation of spatially tuned actions. With this respect, the mouse provides an ideal model system, as a wide range of tools is available for the genetic dissection of the neural networks underlying behavior. Mice, unlike primates, are afoveate and hence lack the primary motive to carry out proper saccadic eye movements. Indeed, in freely moving rodents, vestibulo-ocular reflexes (VOR), rather than saccades, account for nearly all recorded eye movements [17]. Instead of saccades, mice readily perform voluntary head movements toward targets, and fine control of the metric of head movements is essential during natural behaviors, such as exploration and foraging. Hence, the study of head movements in mice provides a tractable, ethologically relevant experimental paradigm for the investigation of spatially tuned actions.

On these premises, within the present work, we sought to determine two things: first, whether a metric for spatially tuned actions exists within the SC of the mouse and, second, the dimensionality in which such a hypothesized motor map operates.

With respect to the first proposition, to date, only very few studies have investigated the SC in unrestrained conditions in rodents [18, 19, 20, 21]. These studies have provided evidence of the involvement of the SC in left versus right spatial choices during goal-directed locomotion [18] and in visual orienting responses [20]. Microstimulations of the rat SC have also implicated the region in producing circling behaviors, with greater stimulation frequencies found to produce greater rates of circling [21]. However, none of these studies has characterized the nature or even the existence of a spatial metric for the decoding of head movements, nor have they attempted to determine the dimensionality of motor tuning in the SC, and therefore, the existence of a metric for the control of three-dimensional (3D) spatially tuned actions in mice still remains unknown.

This takes us to the second aim of this study: the characterization of the dimensionality of the motor map for head movements in mice. The problem of the dimensionality of collicular coding remains a matter of debate also in primates. Indeed, early works support the view that SC neurons decode only two dimensions of the head and eye displacement vector [22, 23, 24] (yaw and pitch) whereas the third torsional dimension (roll) is decoded downstream to the SC [25, 26]. However, more recent studies using head-unrestrained recordings in primates have started to suggest a role of the SC in carrying early signals for the subsequent vector recomposition in the brainstem [27].

With the present work, we characterized the activity of SC neurons in mice while monitoring 3D head displacements in freely moving conditions for the first time in any species. By doing so, we were able to show the existence of a collicular 3D map of head displacements, identifying collicular units tuned individually or conjunctively to all three Eulerian components (yaw, pitch, and roll) of the head-displacement vector. We also characterize the role external landmarks and cell firing properties have in influencing the metric of such a motor map. The establishment of the mouse as a model system to study the metric of head motion also provides a platform for the future genetic investigation of the neural circuits underlying spatially targeted action.

## Results

### Inertial Sensor-Based Approach to Monitor 3D Head Displacements

**Figure 1:**
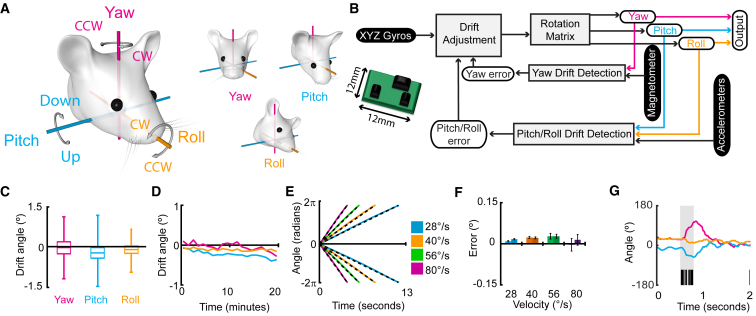
Inertial Sensor-Based Approach to Monitor 3D Head Displacements (A) Cartoon depicting the three Eulerian axes about which head rotations can occur, showing the yaw axis (magenta), pitch axis (cyan), and roll axis (orange). Curved arrows show the definition of rotation directions about each axis. (Right) Separate examples of a clockwise yaw, downward pitch, and counter-clockwise roll rotation relative to an axis-aligned starting position are shown. (B) Cartoon of the inertial sensor and flow schematic showing the implementation of the direction cosine matrix algorithm with the sensor. Gyroscopic information for the three axes is passed through a rotation matrix to determine the orientation of heading. Inputs from accelerometer and magnetometer chips detect drift in the gyroscopic signal before the error is calculated and adjusted for. (C) Box and whisker plots showing the jitter in the sensor system in static regime for each sample recorded at 50 Hz. (D) Line plots depicting the total cumulative drift in the system over 20 min. (E) Line plots showing the sensor output during rotations of the sensor over 360° at four different speeds and two directions (colored lines) and the expected measurement (black dashed line). (F) Bar chart showing the error for each sample at each speed (lighter shades show clockwise rotations; dark shades for counter-clockwise rotations), depicted as mean ± SEM. The error is measured in degrees for each expected degree per temporal bin. (G) Implementation of the sensor aligned with tetrode recordings showing the traces of yaw (magenta), pitch (cyan), and roll (orange) aligned to the bursting activity of a neuron recorded from the SC (black lines represent spikes; highlighted gray area indicates the bursting window).

In order to study the dimensionality of the spatial action map of head movements in the mouse and to overcome the current limitations in the study of head movements in freely moving animals, we developed an inertial sensor fusion-based system, inspired by aeronautic control system theory, for monitoring head rotations in the three Eulerian components (yaw, pitch, and roll; Figure 1A). We then used it alongside *in vivo* chronic electrophysiological recordings to study the spatial tuning of SC neurons recruited during the execution of spontaneous head movements in freely moving mice. The sensor consists of accelerometers, gyroscopes, and magnetometers. Sensor outputs are fed to a direction cosine matrix (DCM) algorithm to provide measurements of head orientation expressed in Euler angles with respect to the Earth reference frame (yaw, pitch, and roll; Figure 1B; STAR Methods). In order to validate the approach, we first tested for the presence of drift and instability of sensor readings and DCM output in a static regime with the sensor still. Data indicate an error in the DCM output of less than ±0.25° per temporal bin (yaw = −0.039° ± 0.001°; pitch = −0.233° ± 0.001°; roll = −0.113° ± 0.001°; Figure 1C). Over the course of the 20-min recording, the total cumulative drift was −0.27°, −0.37°, and −0.15° for yaw, pitch, and roll, respectively (Figure 1D).

As the primary scope of the system is to monitor head displacement in freely moving mice, we also tested the spatiotemporal reliability of the DCM output in a dynamic regime in which the sensor was subjected to step-motor-controlled displacements of various angular velocities. Results indicate an error of the computed displacement of 0.001° ± 0.008° per degree per sample (Figures 1E and 1F). We found there was no difference in the mean error of displacement between velocities (28°/s = 0.01 ± 0.001; 40°/s = 0.02 ± 0.003; 56°/s = 0.03 ± 0.005; 80°/s = 0.005 ± 0.01; *F*_(3,1784)_ = 1.3; p = 0.26), nor was there a difference in errors between direction of motion (clockwise [CW] = 0.01 ± 0.007; counter-clockwise [CCW] = 0.02 ± 0.009; *F*_(1,1784)_ = 0.5; p = 0.47). There was no interaction of error in measurements between direction and speed of motion (*F*_(3,1784)_ = 0.33; p = 0.80; Figure 1F). Together, these data show a faithful representation of rotations with only marginal measurement error at a variety of speeds and directions. Finally, we integrated the sensor and real-time DCM in our recording system in order to monitor, in real time, neuronal activity and vectorial head displacement in freely moving mice (Figure 1G).

### Angular Head Displacements Are Relatively Unconstrained

Studies in primates have revealed that the head does not routinely make use of all three degrees of freedom of motion, a feature formalized by what is known as the Donders’ law and its corollaries. In the case of primates, this dimensionality reduction originates from constraints on the use of the torsional component of motion. Thus, in order to place any potential neural correlates of head rotations into the context of the range of movements carried out by mice, we first sought to reconstruct head rotations to determine whether there exists a constraint in movement about any of the Eulerian axes.

**Figure 2:**
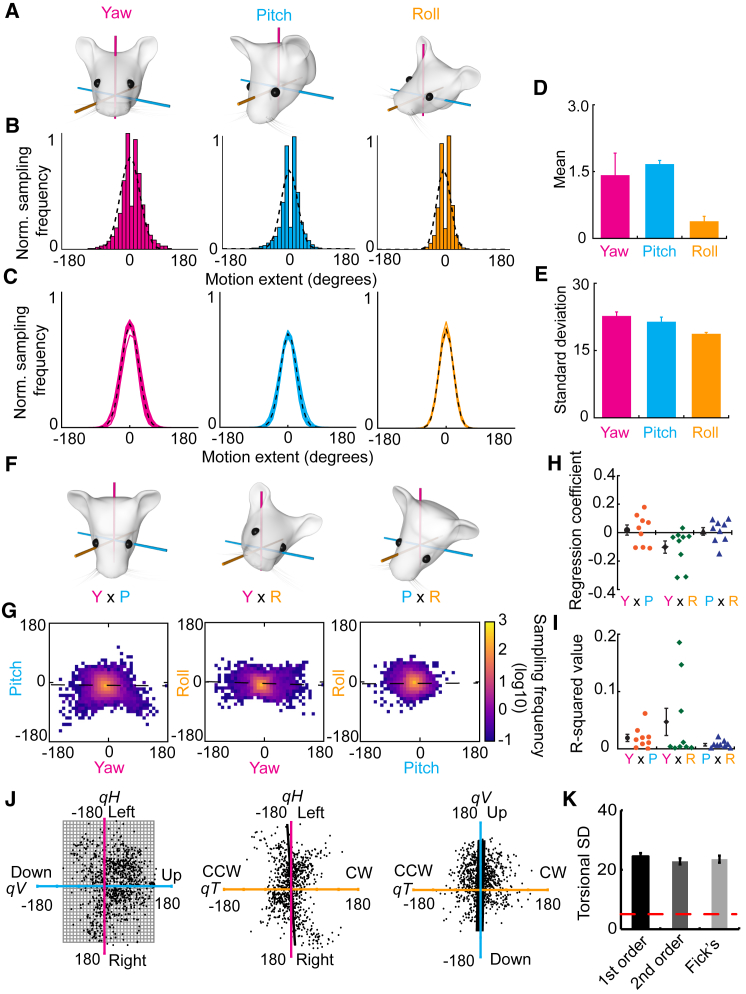
Angular Head Displacements Are Relatively Unconstrained in Mice (A–E) One-dimensional kinematics of head motion. (A) Cartoon depicts examples of rotations around each of the three Eulerian axes. (B) Histograms showing the sampling of movements for each of the axes (yaw, magenta; pitch, cyan; roll, orange) from the trials of one animal. Dashed line shows the Gaussian curve fitted to the histogram of sampled movements. (C) Line plots showing the animal averages (solid lines; n = 9) and population average (black dashed line) of the Gaussian curves fitted to the movement sampling data in yaw (magenta), pitch (cyan), and roll (orange). (D and E) Bar charts showing the mean ± SEM of the center (D) and SD (E) of fitted Gaussian curves from the analyses of nine mice over 96 recording trials. (F) Cartoons showing examples of the three possible pairings of conjunctive head motion. Left to right: yaw × pitch, yaw × roll, and pitch × roll motions are shown. (G) Heatmaps depicting the sampling of head motions for each pair of conjunctive motions taken from the trials (n = 8) of one mouse. Warmer colors represent greater sampling. Black dashed lines depict the output of the regression carried out for this mouse. (H and I) Scatterplots showing the individual regression coefficient (H) and associated R^2^ values (I) for each mouse (n = 9) in each pair of conjunctive motions. The mean ± SEM of the mice is shown to the left of each group of scatterplots in black. (J) Quaternion representation of conjunctive motion sampling from the mouse shown in (G). (K) Torsional SD values taken from the 1^st^ and 2^nd^ order fitting of Listing’s planes and Fick’s plane (bars) compared to previous studies in primates (red dashed line). Data depicted as mean ± SEM. See also Tables S1 and S2.

Utilizing our newly developed inertial sensor, head motion in all three Eulerian axes was recorded from mice (n = 9) during five-minute foraging sessions (n = 96; 11 ± 2 trials per mouse). Our results indicate that angular head displacement amplitudes are normally distributed for all three Eulerian components of motion, with differing ranges of movement for the different Eulerian components (Sigma: yaw = 22.7 ± 0.7; pitch = 21.4 ± 0.7; roll = 18.3 ± 0.2; *F*_*(*2,16)_ = 21.26; p < 0.0001; Figures 2A–2E), with a smaller range of roll movements than yaw (*t*_(8)_ = 5.9; p < 0.001; Figure 2E) and pitch movements (*t*_(8)_ = 4.7; p = 0.002) and no difference between the yaw and pitch axes (*t*_(8)_ = 2.1; p = 0.07). These data show that a significant torsional component is present during spontaneous head movements but that the range of rotations around the roll axis is reduced compared to the other axes.

Moving on, in order to understand whether the torsional component co-varies with the vertical (pitch) or horizontal (yaw) component of the displacement vector, we also characterized the conjunctive nature of these movements (Figure 2F). Two-dimensional head-displacement heatmaps were constructed from the analysis of all motion bouts for each animal (Figure 2G). Comparisons of the slope of the fit linear regression revealed that there was no particular directional bias for the correlation between yaw and pitch (0.018 ± 0.036; t_(8)_ = 0.50; p = 0.63; Figure 2H) or between pitch and roll displacements (0.008 ± 0.027; t_(8)_ = 0.31; p = 0.76). The R^2^ values for each of these comparisons were also low (yaw × pitch = 0.02 ± 0.01; pitch × roll = 0.007 ± 0.002; Figure 2I), indicating that there is very little covariance between conjunctive yaw and pitch and conjunctive pitch and roll rotations. A small covariance of rotations was found for conjunctive yaw and roll movements, in which clockwise yaw movements were more likely to be concomitant with counter-clockwise roll movements (−0.102 ± 0.043; t_(8)_ = −2.36; p = 0.046); however, there remains a wide variety of roll rotations that can occur for any given yaw rotation (R^2^ = 0.05 ± 0.02).

Furthermore, to directly address the issue of Donders’ law conformity and to compare constraints on head motion in mice with those previously described in primates, we expressed head displacement vectors in quaternion space (STAR Methods) in adherence to earlier primate studies [28]. Obedience to the Donders’ law would constrain the tips of the quaternion vectors on a surface. In order to assess this, we fitted a first order, second order, and Fick surface to the extrapolated quaternion data (Figure 2J), an operation conceptually equivalent to fitting a curve to two-dimensional data, and then calculated the torsional SD (Tsd), which is the SD of the data from the surface fit along the torsional axis of this quaternion space. The Tsd of the fit is a quantifiable measure of the adherence to the Donders’ law, with low Tsd indicating adherence and progressively higher Tsd indicating violations of the law [28]. Our data show very large Tsd values, with minimally divergent results between surfaces of different orders (1^st^ order = 24.9° ± 1.2°; 2^nd^ order = 23.5° ± 1.1°; Fick’s = 24.5° ± 1.4°, Figure 2K), highlighting the poor fit of the data to a surface and indicating a poor adherence of head displacements to Donders’ constraints. These values are up to an order of magnitude greater than those reported in primates in spite of the comparable extension of the fitting surfaces between primates and mice (see Tables S1 and S2). Overall, these data indicate that, in freely moving conditions during exploratory behavior, mice exploit all three degrees of freedom of head motion within the boundaries dictated by the mechanical constraints of the head-neck system.

### Tuning of Collicular Neurons to 3D Head Rotations

**Figure 3:**
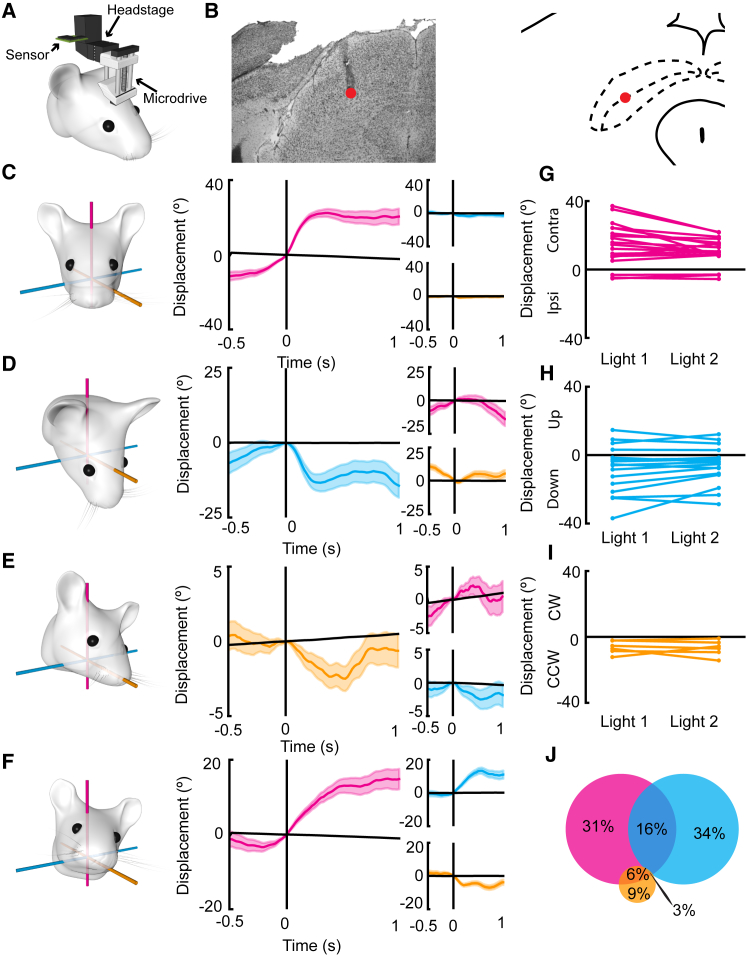
Tuning of Collicular Neurons to 3D Head Rotations (A) Cartoon depicting the attachment of the sensor to the recording system. The sensor was attached to the side of the headstage, which was connected to the implanted microdrive for recording sessions. (B) Example histology showing the photomicrograph of a thionine-stained section (left) and the estimated position taken from the mouse brain atlas [29]. Red dot shows the estimated final position of the tetrodes in the intermediate SC. (C–F) Examples of burst-triggered average analyses (BTA) showing neurons decoding contralateral yaw only (C), downward pitch only (D), counter-clockwise roll only (E), and conjunctive 3D rotations around all three axes—CW yaw, upward pitch, and CCW roll (F). Cartoons on the left depict the resulting motion of the head from each of these neurons, from an axis-aligned starting position. Colored line plots show the burst-triggered average displacements 0.5 s before and 1 s after the onset of bursting for yaw (magenta), pitch (cyan), and roll (orange). Quasi-horizontal black lines depict the mean of displacement angles drawn from shuffled data at each time point. Vertical black lines depict burst onset. Bold colored line depicts mean displacement angle at each time point, and shaded areas depict SEM. (G–I) Comparisons of the resultant motion for cells with yaw tuning (n = 18; G), pitch tuning (n = 17; H), and roll tuning (n = 6; I) for each of the light trials. Note the consistency of tuning across trials. (J) Venn diagram depicting the percentage of motion-tuned cells (n = 32) that are tuned to yaw, pitch, roll, or are conjunctively tuned to yaw and pitch, yaw and roll, or yaw, pitch, and roll. See also Figures S1, S2, S3, and S4.

Having shown that mice make ample use of all three degrees of freedom during spontaneous head movements, we went on to investigate to what extent neurons in the SC are tuned to 3D head rotations. Mice (n = 8) were implanted with tetrode bundles in the intermediate layers of the left SC (Figures 3A, 3B, and S1). Once single units had been isolated, neurons were recorded as mice foraged a square open arena. Each recording session comprised four five-minute recording trials, with two light and two dark conditions (experimental order: light 1 – dark 1 – dark 2 – light 2). Burst-triggered average (BTA) analysis of head motion was carried out for each of the Eulerian components separately (Figures 3C–3F). As each component was analyzed separately, we will henceforth refer to motion tuning to each component as displacement “angles” rather than vectors. This analysis was carried out on bursting events in line with findings in previous studies suggesting that saccades are elicited by the high-frequency bursting of neurons in the SC [10]. Cells were defined as motor tuned based on comparisons to shuffled distributions of mean displacement angles (see STAR Methods); this allowed for us to account for any potential bias in turning directions that may have been exhibited in recording trials.

From 300 isolated neurons, 65.1% ± 7.4% exhibited bursting activity in both light trials. Of these bursting neurons, 16.1% ± 4.4% were consistently tuned to angular head displacements around at least one of the Eulerian components. Of these robustly motion-tuned neurons, 75% were tuned to one component only (yaw = 31.3%; pitch = 34.4%; roll = 9.4%), 21.9% were tuned to two of the three Eulerian components (yaw × pitch = 15.6%; yaw × roll = 6.3%; pitch × roll = 0%), and 3.1% were tuned conjunctively to all three Eulerian components (Figures 3G–3J). The average numbers of bursts elicited by motion-tuned cells were 191 ± 28 (48.6% ± 3.9% of spikes) and 184 ± 32 (47.6% ± 4.4% of spikes) with mean firing rates within bursts of 70.4 ± 2.3 Hz and 71.0 ± 21.2 Hz and mean interspike intervals of 21 ± 0.07 and 21 ± 0.06 ms for the first and second light trials, respectively. We confirmed the contribution of bursting events to the motion tuning of these neurons by comparing the mean displacement angles elicited by spike triggered average (STA) analyses of spikes occurring within bursts and those occurring outside of burst epochs (Figure S2). Cells with yaw tuning revealed a significant loss of absolute tuning angle for spikes occurring outside of bursts compared to those within bursts (within burst = 13.7° ± 2.1°; outside burst = 5.5° ± 1.2°; t_(17)_ = 7.0; p < 0.001; Figure S2A). This was also observed for pitch-tuned cells (within burst = 10.8° ± 2.3°; outside burst = 3.1° ± 1.1°; t_(16)_ = 4.5; p < 0.001; Figure S2B) and roll-tuned cells (within burst = 6.4° ± 1.7°; outside burst = 1.5° ± 0.5°; t_(5)_ = 3.4, p = 0.02; Figure S2C). Together, these results reveal that the relative contribution for spikes occurring within bursts to the average displacement angle is greater than that for spikes occurring outside of bursts.

We next examined the extent, direction, and stability of tuning between light trials (Figure 3G). Neurons tuned to yaw (n = 18) exhibited a preference for tuning contralateral to the recording site. As all animals were implanted in the left hemisphere, this manifested as a preference for clockwise yaw rotations (contralateral: 83.3%; ipsilateral: 16.7%; Figure 3G), with an average extent of tuning (mean displacement angle) of 14.1° ± 1.8°. Comparison between light trials revealed no significant change in tuning between the two trials, with the average change in tuning of −10.9% ± 10.0% of the tuning in the first light trial (t_(17)_ = 0.11; p = 0.30). Neurons tuned to pitch (n = 17) were more likely to be tuned to downward pitch (down: 76.5%; up: 23.5%; Figure 3H), with an average extent of tuning of 10.6° ± 2.1°. There was no significant change in tuning between light trials (−9.4% ± 13.5%; t_(16)_ = 0.62; p = 0.55). All of the roll-tuned neurons (n = 6) recorded exhibited a preference for counter-clockwise rotations (Figure 3I), with an average tuning of 6.5° ± 1.5° and no change in tuning between light trials (percentage change = 15.1% ± 63.0%; t_(5)_ = 0.24; p = 0.82).

Comparison of the within-cell variability of tuning (tuning difference between light trials) and between cell variability (tuning differences between cells) revealed that there is less variability within neurons than between different neurons for yaw- (t_(17)_ = 2.24; p = 0.04; Figure S3A) and pitch-tuned neurons (t_(16)_ = 3.57; p < 0.01; Figure S3B). No effect was observed for roll-tuned neurons (t_(5)_ = 0.14; p > 0.05; Figure S3C). These data indicate that motion-tuned neurons in the SC exhibit preferred angles of displacement that differ between cells, providing support for the presence of a metric for motion tuning in the SC as opposed to a tuning to movement direction alone. This effect could not be explained by different ranges of motion sampling between recording trials (Figures S3D–S3F) for yaw-tuned (R^2^ = 0.11; F_(1,16)_ = 2.01; p = 0.18), pitch-tuned (R^2^ = 0.04; F_(1,13)_ = 0.52; p = 0.48), or roll-tuned (R^2^ = 0.03; F_(1,4)_ = 0.12; p = 0.74) neurons.

Another question, predicated on previous findings that suggest SC activity precedes the onset of motion (for both saccades and head movements) in primates and cats [10, 13], is whether SC neurons in the mouse SC precede angular head displacements. To test this, we calculated the number of bursting events occurring in the 500 ms prior to and following motion onset (Figures S4A–S4D) for each of the motion-tuned cells (n = 32). Comparisons of average *Z* scores of bursting activity across four 80-ms time windows beginning 160 ms and 80 ms prior to motion onset, at motion onset, or 80 ms after motion onset revealed a significant increase in the *Z* score of bursting events over time (F_(2.7,82.7)_ = 10.6; p < 0.001; Figure S4E). Bonferroni corrected paired t tests revealed a significant increase in bursting activity between the first and second time points prior to motion (−160 to −100 ms: *Z* = 0.007 ± 0.007; −80 to −20 ms: *Z* = 0.05 ± 0.01; t_(31)_ = 4.19; p = 0.001), thus providing evidence for the presence of motor-tuned bursting activity just prior to motion onset. There was no difference in bursting activity between the last time point prior to motion onset and the time point immediately following motion onset (0–60 ms: *Z* = 0.06 ± 0.01; t_(31)_ = 0.44; not significant [n.s.]) or between the two time points following motion onset (80–140 ms: *Z* = 0.04 ± 0.01; t_(31)_ = 2.20; n.s.).

Together, the data presented show that the bursting activity of neurons in the intermediate and deep layers of the SC is correlated with, and potentially triggers, 3D dimensional rotations of the head in mice and that the preferred displacement angles of these neurons remain stable between trials.

### Firing Rate Modulates Angular Velocity, but Not Displacement Angle

**Figure 4:**
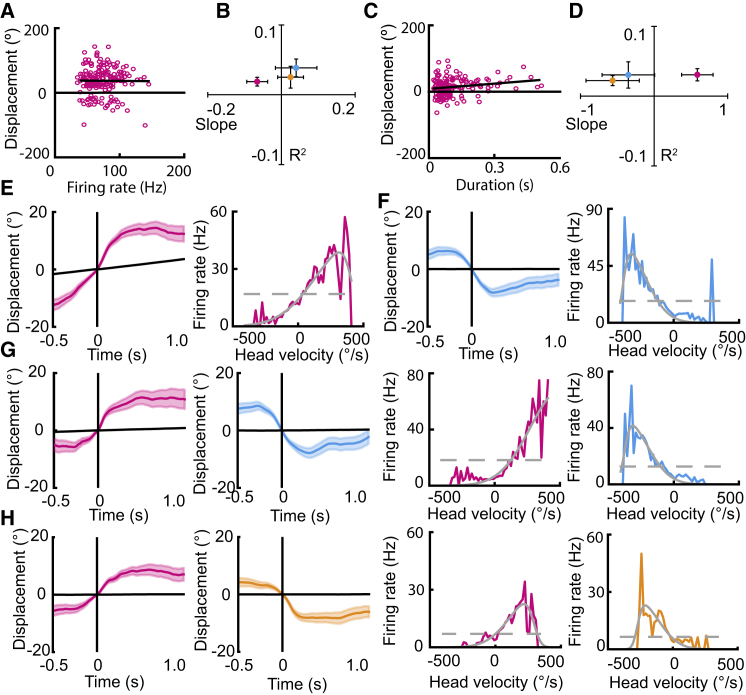
Firing Rate Is Modulated by Angular Velocity, but Not Displacement Angle (A–D) Burst rate and duration are not related to tuning. (A) Scatterplot showing an example of the relation between firing rate and displacement angle for one yaw-tuned cell. Black line shows fit regression line. (B) Scatterplot showing the mean ± SEM of the slope and R^2^ values of the regressions carried out between burst rate and displacement angles for motion-tuned neurons with yaw (n = 18), pitch (n = 17), or roll (n = 6). Note the low R^2^ values. (C) Scatterplot showing the relationship between burst duration and displacement angle for one yaw-tuned cell. (D) Scatterplot showing the mean ± SEM of R^2^ values and regression coefficients for the regression carried out between burst duration and displacement angles for motion-tuned neurons with yaw (n = 18), pitch (n = 17), or roll (n = 6). (E–H) Velocity tuning of four representative cells tuned to (E) yaw, (F) pitch, (G) yaw and pitch, and (H) yaw and roll. (Left plots) Burst-triggered average plots for neurons display the mean ± SEM displacement in the 0.5 s prior to and 1.0 s after bursting onset (vertical line), only shown for the component in which burst-triggered average analyses revealed motion tuning. Horizontal lines show the mean of the shuffled distribution for the cell. (Right) Line plots for the same cells show the increase in firing rate with angular head velocity for the component in which cells are tuned. Grey lines show the results of model fitting for the constant model (dashed line) and skewed Gaussian model (solid line). See also Figure S5.

We next sought to determine the firing characteristics of motion-tuned neurons that may define their tuning. First, we tested whether the firing rates of bursting events were correlated with the displacement angle of neurons. We did not find any consistent correlation (across both light trials) between the firing rate during each burst epoch and the resultant head displacement for the Eulerian component to which each neuron was tuned (Figures 4A and 4B; yaw tuned: R^2^ = 0.02 ± 0.006, β_1_ = −0.07 ± 0.03; pitch tuned: R^2^ = 0.04 ± 0.01, β_1_ = 0.04 ± 0.06; roll tuned: R^2^ = 0.03 ± 0.02, β_1_ = 0.02 ± 0.04). Nor did we find a consistent correlation between the duration of bursting and motor displacements (yaw tuned: R^2^ = 0.03 ± 0.01, β_1_ = 0.59 ± 0.21; pitch tuned: R^2^ = 0.03 ± 0.02, β_1_ = −0.35 ± 0.36; roll tuned: R^2^ = 0.02 ± 0.01, β_1_ = −0.56 ± 0.36); only one neuron (yaw tuned only) exhibited a correlation between duration and tuning on both light trials for yaw (Figures 4C and 4D; light 1: R^2^ = 0.18, β_1_ = 1.53, p < 0.001; light 2: R^2^ = 0.14, β_1_ = 1.56, p < 0.001).

While firing rate and burst duration were not correlated with the displacement angle associated with SC neurons, the firing rates of neurons were found to be tuned to angular head velocity (AHV). Normalized firing rates were calculated for all motion-tuned SC neurons at a range of velocities between −500°/s and 500°/s (Figures 4E–4H). A constant model (which predicts no modulation of firing rate by angular head velocity) and a skewed Gaussian model (predicting a directional increase in firing rate with velocity) were fit to the data and compared using Bayesian information criterion scores (BICs). A cell was considered tuned if their BIC was 10 or more points lower [30] for the skewed Gaussian model than the constant model and in the direction of the cell’s average displacement angle. Of cells with a yaw-tuned component (n = 18), 72.2% passed this criterion, whereas 46.7% and 50.0% of pitch- (n = 17) and roll-tuned (n = 6) cells, respectively, were considered to have firing rates modulated by angular head velocity (Figure S5). We note that around 44% of motion-tuned neurons did not exhibit any angular head velocity tuning, indicating that velocity tuning is not necessary for the tuning of SC neurons to displacement angles of the head.

Taken together, the absence of correlation between firing rate or burst duration and the produced motor displacement, alongside the modulation of motion-tuned cell firing rates by angular head velocity, suggest that the identity of motion-tuned neurons, rather than the modulation of their activity state, determines the extent of the produced angular displacement but that the speed at which this displacement is reached can be rate dependent.

### Tuning Occurs in the Absence of Visual Cues

**Figure 5:**
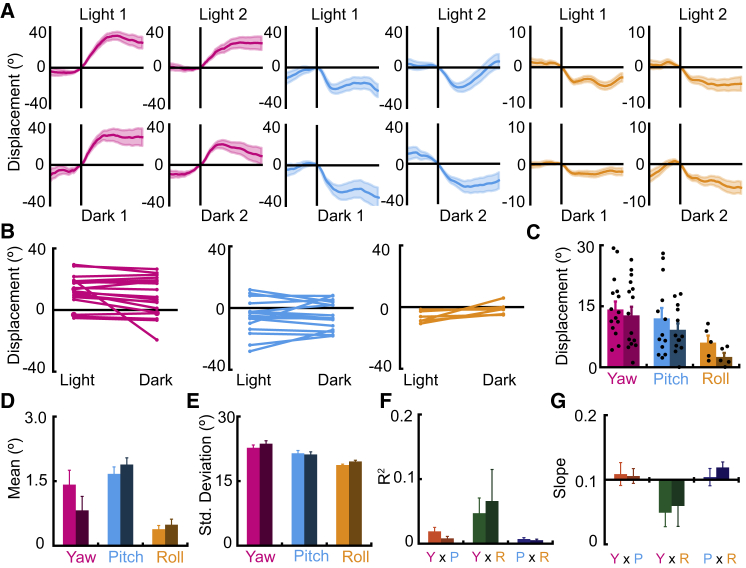
Light-Independent Tuning of SC Neurons (A) Line plots showing burst-triggered averages of head displacements for neurons decoding yaw (left), pitch (middle), or roll (right) in light trials (top) and dark trials (bottom). Note the similarity of tuning between light and dark trials. (B) Comparisons of the mean displacement angles in light and dark trials. Note that not all neurons maintain tuning in dark conditions. (C) Bar (mean ± SEM) and scatterplots depicting the absolute displacement angle of neurons in light trials (light shaded bars) and dark trials (darker shaded bars). For clarity, only neurons that maintained the same direction of tuning in light and dark trials are shown in (C). (D) Gaussian curves were fit to the sampling frequencies of head displacement events for dark trials (darker shades) as well as light trials (lighter shades, also shown in Figures 2D and 2E) for each of the three Eulerian axes. There was no effect of condition (light versus dark) on the mean of the fit Gaussian curves. (E) There was an effect of condition on the SD of the fit Gaussian curve, as well as an interaction between Eulerian component and condition, shown by an increased range of sampling in the yaw and roll axes in dark conditions. (F and G) The results of regression analyses for conjunctive movements in light (lighter shades) and dark (darker shades) for each pair of conjunctive movements. There was no effect of condition on either the associated R^2^ values (F) or regression coefficient (G) for any of the conjunctive pairings. In (D)–(G), motion sampling in darkness depicted as mean ± SEM.

One question of interest regarding the motion tuning of SC neurons asks how sensory information is integrated in the production of elicited motion vectors. To this aim, we also carried out recordings in two five-minute trials in darkness (Figures 5A–5C). Of the motion-tuned neurons described above, 88.9% of neurons with tuning to yaw displacements exhibited an average tuning across the two dark trials in the same direction as in the average of the two light trials. This was similar for pitch- (76.5%) and roll-tuned neurons (83.3%).

In more detail, yaw-tuned neurons that exhibited the same direction of tuning in light and dark trials (n = 16) showed no change in their associated angular displacement in dark conditions (percentage shift between conditions: −1.9% ± 4.1%; *t*_(15)_ = 1.8; p = 0.09; Figures 5B and 5C), with 37.5% of cells exhibiting an increase in the absolute displacement angle in darkness and 62.5% exhibiting a decrease. Neurons exhibiting the same tuning direction to pitch displacements in light and dark trials (n = 13) exhibited no change in their associated angular displacement (percentage shift = 21.1% ± 58.0% of light trial tuning; *t*_(12)_ = 1.31; p = 0.21) with an increase in absolute displacement angle for 61.5% of neurons and a decrease in 38.5% of neurons. 83.3% of roll-tuned cells (n = 5) exhibited a decrease of absolute angular displacement, with an average loss in tuning of 50.0% ± 19.6% of the light trial tuning (*t*_(4)_ = −2.54; p = 0.06). There was no change in the number of bursting events between light and dark trials that could explain the change in tuning between conditions (light: 193 ± 29; dark: 225 ± 38; t_(31)_ = 1.5; p = 0.15). Overall, there was no decrease in the range of motions in darkness that could explain a change in tuning (Figures 5D–5G), with animals carrying out, on average, a wider range of motions in darkness (*F*_(1,8)_ = 24.6; p = 0.001) with SDs of the fit Gaussian curves increasing for yaw (light = 22.72 ± 0.66; dark = 23.68 ± 0.67; t_(8)_ = 3.69; p = 0.006) and roll displacements (light = 18.74 ± 0.18; dark = 19.55 ± 0.27; t_(8)_ = 4.78; p = 0.001). There was no effect of condition on the range of pitch movements (light = 21.44 ± 0.65; dark = 21.19 ± 0.6; t_(8)_ = 0.63; p = 0.55). Comparisons of conjunctive motions between dark and light trials (Figures 5F and 5G) revealed no effect of light condition on either the R^2^ value (*F*_(1,8)_ = 0.04; p = 0.84) or slope (*F*_(1,8)_ = 0.89; p = 0.37) of the fit regressions, showing that the absence of light does not change the relationship between pairs of Eulerian components during conjunctive motions.

Taken together, these data indicate that motion-tuned neurons in the SC can maintain tuning for all Euler angles in the absence of light but that the accuracy of the tuning for some neurons is diminished without the presence of visual cues and/or optic flow information.

### Tuning Is Independent of Allocentric Heading

**Figure 6:**
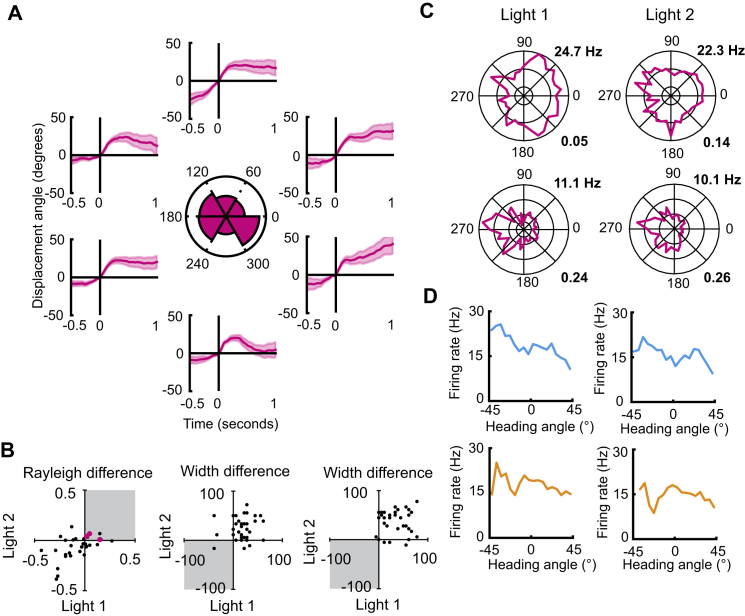
Motion Tuning Is Independent of Landmark Cues (A) BTA plots of a motion-tuned cell with a preference for clockwise yaw shown at six azimuthal headings (60° bins). Polar plot shows the dwell-time-normalized number of bursts in each bin. Line plots show the BTAs of the cell at each of these headings. Note the similarity in tuning. (B) Scatterplots showing the difference in tuning between recording trials and shuffled distributions for azimuth (left), elevation (middle), and bank (right) for each light trial. Data are shown for all motion-tuned cells (n = 32). For azimuth tuning, Rayleigh vector scores were compared—cells with consistently higher Rayleigh scores than 95% of the shuffled distribution (gray box) and a consistent preferred firing direction (within 30°) were considered to be modulated by azimuth heading (pink dots show tuned cells). Tuning width was compared for elevation and bank—only cells with tuning widths less than 5% of the shuffled distribution in both light trials (gray box) were considered to be modulated by elevation or bank heading. None of the motion-tuned cells were modulated by elevation or bank. (C) Polar plots of a non-azimuth-modulated cell (top) and an azimuth-modulated cell (bottom) for both light trials. (D) Examples of the lack of modulation in pitch (top) and roll (bottom) of one cell for both light trial 1 (left) and light trial 2 (right). See also Figure S6.

Further to our recordings in darkness, we tested whether the activity of motion-tuned SC neurons is independent of heading relative to external landmarks (allocentric heading). In order to do this, we determined whether the firing rates of displacement-tuned neurons (n = 32) were modulated by allocentric heading in each of the three Eulerian axes separately (Figure 6). Headings within allocentric coordinates are referred to as azimuth, elevation, and bank for the yaw, pitch, and roll axes, respectively. We first tested for modulation of firing rate by azimuth heading. An example of burst-triggered analyses of one yaw-tuned cell for six 60° bins of allocentric azimuth heading is shown in Figure 6A. Rayleigh vector scores were calculated to test for tuning of firing rates to azimuth and compared to distributions drawn from a random shuffled distribution. Of the motion-tuned neurons, three exhibited Rayleigh vector scores greater than 95% of the respective shuffled distribution in both light trials and maintained a consistent preferred heading angle (within 30°) across the two trials (Figures 6B and 6C). For these units, the associated Rayleigh vector scores indicated a low level of modulation of firing rate by heading direction (light 1 = 0.27 ± 0.02; light 2 = 0.3 ± 0.04) as compared to a widely used threshold for the definition of head direction cells of 0.4 [31, 32].

Next, we tested the tuning to elevation and bank. As the full range of possible directions was not sampled for pitch or roll, a non-circular approach to analysis was taken. Briefly, the tuning widths of cells were compared to shuffled distributions and only cells with tuning widths less than 5% of the shuffled distributions were considered to be modulated by elevation or bank. All motion-tuned cells failed to meet the criteria for non-uniformity that would indicate a directional modulation by either elevation or bank heading (Figures 6B and 6D).

We further tested for allocentric heading in all three axes of heading for the full sample of recorded neurons (n = 300; Figure S6). We found that 3.1% of neurons exhibited modulation by azimuthal heading, 0.6% of neurons were modulated by elevation, and none were modulated by bank (Figures S6A–S6C). Again, none of the azimuth-modulated cells exhibited Rayleigh vector scores above 0.4 in both light trials (mean ± SEM: light 1 = 0.22 ± 0.02; light 2 = 0.24 ± 0.04), further supporting the notion that neurons in the SC do not exhibit the sort of allocentric tuning that is canonically seen in head direction cells, but instead a small proportion of neurons exhibit a low-level modulation of their firing rates by azimuth (Figures 6D–6F). Furthermore, by temporally shifting spike times by five temporal bins (20 ms) both before and after the recorded spike times, we found that azimuth-modulated SC neurons did not exhibit anticipatory or delayed azimuth modulation (F_(2.8,25.3)_ = 0.53; p = 0.65; Figure S6G) [33, 34].

These data show that the firing rate of motion-tuned neurons in the SC is not tuned to allocentric heading in any one of three Earth-referenced heading directions but that, in a small proportion of neurons, there is a low-level modulation of firing rate by allocentric heading. Together, this indicates that the tuning of neurons in the SC to 3D head displacements is egocentric in nature and is largely independent of landmark cues.

## Discussion

In the present study, we set out with the aim of translating the study of spatially tuned actions into a genetically amenable animal model. With this in mind, we sought to determine the existence and dimensionality of a metric for spatially tuned actions in mice.

By studying the extracellular electrophysiology of SC neurons alongside the recording of 3D head displacements in freely moving mice using our head-mounted inertial sensor, we identified collicular units tuned for head rotations in all three Eulerian components. This is the first time that units tuned for the metric of head motion were revealed in rodents and, to the best of our knowledge, the first time that 3D-tuned collicular neurons were identified in any species. Indeed, while previous studies in primates and cats have provided evidence for the role of the SC in encoding head rotations [35, 36], the SC had been suggested to encode only two of the three dimensions of head movements, namely yaw and pitch [22, 23, 24], with the torsional (roll) component being decoded downstream to the SC [25, 26]. Hence, while the present study supports the existence of a conserved control network for the metric of head movements at the level of the SC, it also highlights a divergence with respect to the dimensionality of such a metric, which appears to be two-dimensional in primates and three-dimensional in mice. It is possible that differences in the dimensionality of the collicular tuning for head motion simply reflect differences in the behavioral constraints between previous primate studies and the present work. Indeed, whereas the present work studies head motion during unrestrained naturalistic foraging, previous studies used primates trained to solve two-dimensional visually guided tasks. Hence, the spatially constrained behavior highlighted in these early works may simply reflect a behavioral strategy aimed at optimizing task performance rather than a low dimensionality nature of the collicular signal. In line with this idea, when we compared the behavioral constraints in the three dimensions between our study and previous studies, we found that the freedom of movements in the torsional dimension is up to an order of magnitude greater in mice than in primates. Further work in primates in head-unrestrained conditions or, ideally, in freely moving conditions might clarify whether this full dimensionality of SC motor tuning is indeed a peculiarity of the mouse model or whether it is also present in primates. Conversely, further work in mice in which animals are trained to reach spatially defined points would provide evidence on whether constraints on the torsional dimension are specifically implemented for target-directed movements. It is also important to note that more recent work in primates also begins to highlight the existence of an early collicular signal for the subsequent full 3D vector recomposition in the brainstem [27].

Another feature of the nature of collicular coding for head displacements uncovered in this study concerns the invariance of the head angular displacement with respect to firing rate or burst duration of motor-tuned collicular neurons. The identity of the motion-tuned neurons, rather than their firing rate, determines the amplitude of head displacements. At the same time, we also show that the firing rate of many motion-tuned neurons correlates with the angular head velocity of the produced displacements, suggesting that the firing rates of motion-tuned SC neurons can drive the speed at which selected motion vectors are reached, but not the actual motion vector that is selected. In essence, SC neurons operate as digital controllers with respect to vector selection but can also operate as rate-dependent analog controllers with respect to speed selection. While we note that not all motion-tuned SC neurons exhibit angular head velocity tuning, the presence of such tuning in ∼50% of these neurons may point to additional control of reorientation in the SC that may be of particular use in dynamic behaviors, such as tracking or chasing moving objects, whereas non-angular head velocity-tuned neurons may be better suited to providing the more general command for amplitude of motion. The future dissection of the relative contribution of these two classes of motion-tuned SC neurons to reorienting behaviors may uncover a diverse motor coding in SC that underlies the flexible control of different movement types (e.g., ballistic versus tracking behaviors) that occur in different behavioral contexts.

Interestingly, to the best of our knowledge, this is the first time that such angular head velocity tuning to the three Eulerian components has been seen outside of the hindbrain in rodents, and it is worth noting that, outside of the SC, similar 3D tuning of angular head velocity may also be needed. For example, in the head direction cell system, when animals move over complex 3D structures, head direction-cell-preferred firing directions are updated by pitch and roll rotations of the head around the gravity vector, allowing animals to maintain an accurate Earth-horizontal-referenced orientation, irrespective of the orientation of their locomotor surface [6]. Given that the head direction system is hypothesized to require angular head velocity signals to shift head direction cell activity around a ring attractor [37], it is at least plausible that the angular head velocity signals produced in the SC might be made available to the head direction cell system. A point to note with regards to the tuning of SC units to angular head velocity is that the current study did not set out to test the direction of causality between firing rate and angular head velocity. We therefore cannot be certain whether increased firing rates drive higher velocity movements or whether the firing rate of SC motion-tuned neurons is modulated by sensory feedback relayed from the vestibular system. However, based on previous evidence that the rate of circling behaviors increases with higher stimulation rates [21], it seems more likely that the firing rate of motion-tuned SC neurons plays a causal role in determining movement velocity.

Regarding the sensory involvement in the computation of head-displacement vectors, it seems likely that some form of sensory feedback would be required to ensure that the metric of motion is faithfully obeyed. The maintenance of tuning for the majority of neurons in darkness as well as the comparable behavior between light and dark conditions rules out the necessity of visual input in the computation of head motion vectors. However, it remains interesting that, whereas most neurons maintain tuning in darkness, a subpopulation of neurons loses tuning. One possibility is that the SC contains subpopulations of motion-tuned neurons that depend differently on visual input. This may also be true of input from other sensory modalities, such as the vestibular system. Indeed, previous research in rodents indicates that the intermediate layers of SC serve as a point of convergence of multiple sensory modalities, with rodent SC neurons tuned to tactile, auditory, and visual stimuli [19]. Some of these neurons also exhibit bipartite or even tripartite tuning to different sensory modalities. It is therefore plausible that subpopulations of motion-tuned neurons with the same preferred displacement vectors are influenced by different sensory modalities, allowing for the maintenance of spatially tuned actions in the absence of one or more sensory inputs. Future work, using approaches targeted at specific sensory networks, will be needed in order to disentangle the relative role of the sensory modalities in the execution of spatially tuned actions—a goal made more achievable with the establishment of the mouse as a model system for studying such behaviors.

A question remains regarding the reference frame used by motion-tuned SC units. Answering this question is of interest, as it could indicate whether the SC defines spatially tuned actions in an Earth-centered space or head-centered space. The divergence between the predictions of these two reference frames increases at more extreme roll and pitch values. In the present study, the number of bursting epochs occurring at angles greater than ±45° in pitch or roll was very low, and as such, there was very little divergence in predictions between the two reference frames (data not shown). Future work could address this issue directly by training mice to begin targeted motions from heavily offset pitch or roll headings and thus increasing the divergence between the calculated rotations in the two reference frames.

Another issue of interest for future studies regards the topographic nature of motion vectors elicited by SC neurons. Studies in primates and cats reveal a topographic organization of motor activity in the elicitation of eye and head movements. Microstimulations have revealed a retinotopic map for saccades and head movements [10, 38], in which stimulation of more posterior regions of intermediate and deep SC elicit higher amplitude contralateral horizontal movements, whereas medial stimulations elicit upward movements and lateral stimulations elicit downward movements. Determining whether a similar retinotopy exists for yaw and pitch rotations of the head in the mouse will likely be best addressed with a site-by-site examination of the motor behaviors elicited by stimulation, either electrically or optogenetically, in the mouse.

Finally, it seems valuable to discuss the possible cognitive role that the networks responsible for the execution of spatially tuned head displacements described in this work might have with respect to spatial encoding. It has been suggested that motor-displacement vectors, such as those recruited by the eye-head-reach systems, might also serve a cognitive function by defining a relational transformation map that determines object location within the peripersonal space [39, 40], in line with a motor-centric model of space encoding [41]. Within this conceptual framework, the work presented here paves the way not only to the dissection of networks responsible for the generation of spatially tuned head movements but also to the study of peripersonal space encoding in mice. In prospective, the genetic amenability of the mouse model opens a new frontier in both of these directions.

## STAR★Methods

### Key Resources Table

**Table:** 

REAGENT or RESOURCE	SOURCE	IDENTIFIER
**Chemicals, Peptides, and Recombinant Proteins**		

Thionin Acetate Salt	Sigma Aldrich	Product #861340
Platinum Chloride	Sigma Aldrich	Product # 206091
Histoclear	National Diagnostics	Catalog # HS-200

**Experimental Models: Organisms/Strains**		

Mouse: C57BL/6JOlaHsd	Envigo	Order Code: 057

**Software and Algorithms**		

Prism 7	Graphpad	https://www.graphpad.com/scientific-software/prism/
Python 2.7 (anaconda 4.2.0 distribution)	Continuum Analytics	https://www.continuum.io/downloads
Tint	Axona	Product Number: Comp/TINT01, http://axona.com/products
Arduino	Arduino	http://www.arduino.cc
Processing 3	Processing	Processing.org
Custom Python Scripts	This paper	N/A

**Other**		

Microwire (17μm, platinum iridium)	California Wire Company	Product code 100167, http://www.calfinewire.com/datasheets/100167-platinum10iridium.html
NanoZ plating equipment	Multichannel Systems	nanoZ, http://www.multichannelsystems.com/products/nanoz
Recording system (pre-amp and system unit)	Axona	Product number: DacqUSB/32, http://axona.com/products

### Contact for Reagent and Resource Sharing

Further information and requests for resources and reagents should be directed to and will be fulfilled by the Lead Contact, Marco Tripodi (mtripodi@mrc-lmb.cam.ac.uk).

### Experimental Model and Subject Details

#### Animal strains

C57BL/6 wild-type (WT) mice were used for all of the experiments. All procedures were conducted in accordance with the UK Animals (Scientific procedures) Act 1986 and European Community Council Directive on Animal Care. Subjects were nine male C57BL/6Ola mice aged 10-14 weeks at the time of surgery. One mouse was not included in tetrode recordings because of an error in the implantation of the microdrive mechanism. After surgery mice were individually housed to prevent damage to implants. Lighting was set to a reversed light dark cycle, with simulated dawn and dusk at 7 pm and 7 am, respectively. After a seven-day post-surgery recovery period the animals were placed on a restricted diet sufficient to maintain 85% of their free-feeding weight

### Method Details

#### Design and implementation of inertial sensors

The Direction Cosine Matrix (DCM) algorithm was used to model the orientation of the head of the mouse. Inputs from gyroscopes, accelerometers and magnetometers were fed into the DCM algorithm to provide a measurement of orientation, relative to the earth’s magnetic field (magnetometer) and the direction of gravity (accelerometer), expressed by the Euler angles (yaw, pitch and roll). The DCM algorithm calculates the orientation of the sensor in respect to the earth reference frame by using rotation matrices. The rotation matrices describe the three consecutive rotations needed to describe the orientation.

The gyroscopes are the primary sensors used to calculate the orientation of the system. However, gyroscopes have different offsets that cause angular drift over time after integration. The accelerometers and magnetometers are then used to provide orientation references to detect the gyroscope offsets and to adjust the error through a proportional plus integral feedback loop. A step of renormalization was also applied to correct numerical errors that affect the orthogonality conditions of the rotation matrix. The inertial sensor system was developed so that it could be fixed to the head of the mouse, and as such descriptions of the workflow of the sensor system will be described with reference to the heading direction of the mouse. We will first introduce rotation matrices and how they are used to extract Euler angles, before describing the use of gyroscopes to retrieve angular head velocity signals and the use of the rotation matrices in computing Euler angles over time. Finally, we will detail the numerical correction on the rotation matrix and the implementation of outputs from the other sensors to ensure the reliability of sensor output.

#### System orientation - Rotation Matrices

In order to describe movements of the head of the mouse, we chose an Earth-fixed coordinate system as the inertial frame of reference. Axes were chosen so that the x axis points north, the y axis points east and the z axis points downward. From that, we defined the Euler angles yaw, pitch and roll which represent the clockwise rotations to their respective axis. Thus, yaw ψrepresents rotation about the z axis, pitch θ represents rotation about the y axis, and roll ϕ represents rotation about the x axis.

The head position of the mouse is given by the sensors and was represented as a new coordinate system called the head frame. The x axis of the head frame points out the nose of the mouse, the y axis points out of the right of the head, and the z axis points out of the bottom of the head. The head frame was obtained by performing the rotations from the inertial frame of reference by the anglesψ, θ and ϕ.

Mathematically, the three rotations can be represented by a rotation matrix R which expresses how the vector measured in the inertial frame of reference is rotated to the head frame. Hence, if $V_{I}$ and $V_{H}$ respectively represent a vector in the inertial frame and in the head frame, then $V_{H}=RV_{I}$. We can then decompose the rotations in three successive rotations from the inertial frame. First we rotate the z axis of the inertial frame by the yaw angle. This rotation gives us a temporary head frame (head-1 frame). Then we rotate the y axis of the head-1 frame to obtain a new temporary frame (head-2 frame). Finally, the rotation of the x axis of the head-2 frame gives us the real head frame. The rotation matrices given by yaw, pitch and roll are given below.

$$R_{z}\left(\psi \right)=\left(\begin{array}{ccc} \cos\;\psi & \sin\;\psi & 0\\ -\sin\;\psi & \cos\;\psi & 0\\ 0 & 0 & 1 \end{array}\right)$$

$$\;R_{y}\left(\theta \right)=\left(\begin{array}{ccc} \cos\;\theta & 0 & -\sin\;\theta \\ 0 & 1 & 0\\ \sin\;\theta & 0 & \cos\;\theta \end{array}\right)$$

(1) $$R_{x}\left(\phi \right)=\left(\begin{array}{ccc} 1 & 0 & 0\\ 0 & \cos\;\phi & \sin\;\phi \\ 0 & -\sin\;\phi & \cos\;\phi \end{array}\right)$$

In fact, we can directly obtain the complete rotation matrix for moving from the inertial frame to the head frame by multiplying the yaw, pitch and roll matrices:

(2) $$R\left(\phi ,\theta ,\psi \right)=R_{x}\left(\phi \right)R_{y}\left(\theta \right)R_{z}\left(\psi \right)$$

Rotation matrices are not commutative so the ordering of these three rotation matrices depends on the order in which the three rotations are applied. Other ordering would give different position results. In our case, we use the rotation matrix $R_{xyz}$.

This rotation matrix is given by:

(3) $$R_{xyz}=\left(\begin{array}{ccc} \cos\;\theta \;\cos\;\psi & \cos\;\theta \;\sin\;\psi & -\sin\;\theta \\ \sin\;\phi \;\sin\;\theta \;\cos\;\psi -\cos\;\phi \;\sin\;\psi & \sin\;\phi \;\sin\;\theta \;\sin\;\psi +\cos\;\phi \;\cos\;\psi & \sin\;\phi \;\cos\;\theta \\ \cos\;\phi \;\sin\;\theta \;\cos\;\psi +\sin\;\phi \;\sin\;\psi & \cos\;\phi \;\sin\;\theta \;\sin\;\psi -\sin\;\phi \;\cos\;\psi & \cos\;\phi \;\cos\;\theta \end{array}\right)$$

We will call this rotation matrix R. $R_{ij}$ is defined as the coefficient of the i^th^ row and j^th^ column of the rotation matrix. Thus, the three Euler angles can be deducted from the rotation matrix:

$$\;\phi =\text{atan}2\left(R_{23},R_{33}\right)$$

$$\theta =\;-\arcsin\left(R_{13}\right)$$

(4) $$\psi =\text{atan}2\left(R_{12},R_{11}\right)$$

One property of rotation matrices is that their inverse $R^{-1}$ is equal to their transpose $R^{T}$. This property is utilized later on in the computation of the DCM algorithm and correction of the yaw output. Each of the columns of the rotation matrix are orthogonal to each other and each column’s magnitude is equal to 1.

The presence of a particular case, named Gimbal Lock, should be noted. This case occurs when the head reaches a specific orientation which cannot be described distinctly by the three Euler angles. Since Gimbal lock depends on the order of rotations used, it occurs in our case when pitch is at ± 90 degrees (angles very seldom visited by the mouse). Indeed, considering this configuration, we cannot differentiate between the part that either yaw or roll played in determining the final position of the movement.

#### Gyroscope

Gyroscope measurements represent the angular velocity (the derivation) of each Euler angle. ω represents the angular velocity given by the gyroscope data where:

(5) $$\;\omega =\;\left(\begin{array}{c} \omega_{x}\\ \omega_{y}\\ \omega_{z} \end{array}\right)=\;\left(\begin{array}{c} \frac{\partial \phi }{\partial t}\\ \frac{\partial \theta }{\partial t}\\ \frac{\partial \psi }{\partial t} \end{array}\right)\;$$

One method with which to calculate the Euler angles would be to integrate the angular velocity of each axis over time, however, while the results of this conversion are reliable concerning fast changes of the angles (high frequencies) the data from gyroscopes contain bias which results in drift over time.

#### Implementation of the Direct Cosine Matrix

We show above that we can extract the Euler angles from the rotation matrix. In this step we link the rotation matrix to the primary source of our measurement, the gyroscopes.

A kinematic property of any point represented by a vectorr, rotating around an axis with an angular velocity vector ω  is characterized by:

(6) $$\frac{dr\left(t\right)}{dt}=\omega \left(t\right)\times r\left(t\right)=\;{\text{Ω}}_{\times }\left(t\right)\;r\left(t\right)$$

Where ${\text{Ω}}_{\times }$ represents the antisymmetric matrix related to the cross product (×) of the angular velocity vector:

(7) $${\text{Ω}}_{\times }=\left(\begin{array}{ccc} 0 & -\omega_{z} & \omega_{y}\\ \omega_{z} & 0 & -\omega_{x}\\ -\omega_{y} & \omega_{x} & 0 \end{array}\right)$$

However, Equation 6 is only valid if both vector ω  and r are measured in the same frame of reference. Our aim is to track the axes of the head frame in the inertial frame but the gyroscope data are given in the head frame. From the point of view of the head frame, it is the inertial frame which rotates at an angular velocity of ω  but in the opposite direction. The angular velocity vector representing the rotation of the inertial frame compared to the head frame is then –ω. In that case, if the vector r is in the inertial frame and because the cross product is anticommutative, we can transform Equation 6 into:

(8) $$\frac{dr\left(t\right)}{dt}=r\left(t\right)\times \omega \left(t\right)$$

From Equation 8, if we replace the vector r by the three axes of the head frame viewed in the inertial frame which are represented by the three column vector from the transpose of the rotation matrix, we obtain the following equation:

(9) $$\frac{dR^{T}\left(t\right)}{dt}=R^{T}\left(t\right){\text{Ω}}_{\times }\left(t\right)$$

Because the sampling frequency of the sensor is high (50Hz), we can assume that the rotation matrix will not change appreciably between every time step of 20 ms. We therefore use the following approximation:

(10) $$\frac{R^{T}\left(t+dt\right)-R^{T}\left(t\right)}{dt}≅\frac{dR^{T}\left(t\right)}{dt}$$

From Equation 9 and 10, we obtain the main equation used to update the rotation matrix over time from gyroscope signals:

(11) $$R^{T}\left(t+dt\right)=R^{T}\left(t\right)\left(\begin{array}{ccc} 1 & -\omega_{z}dt & \omega_{y}dt\\ \omega_{z}dt & 1 & -\omega_{x}dt\\ -\omega_{y}dt & \omega_{x}dt & 1 \end{array}\right)$$

The Euler angles can be extracted from the updated rotation matrix every time step as seen in Equation 4.

#### System corrections

The rotation matrix is linked to the angular velocity but corrections need to be applied to the raw data from the gyroscope. This is because numerical errors may accumulate over time due to the approximation made in Equation 10. Drift also accrues from the gyroscope output, and this is corrected using output from the accelerometers and the magnetometer. We next show how to correct these errors.

#### Normalization

Numerical errors affect the orthogonality conditions of the rotation matrix over time, which left uncorrected for would lead to an incorrect representation of the head frame axes. To prevent this, we enforced the orthogonality conditions of the matrix. The respective columns of the rotation matrix are defined as three vectors, X, Y, and Z.

First, we defined the error between the first and second columns of the rotation matrix as the dot product between those two vectors. Indeed, the orthogonality of the rotation matrix supposes that its column vectors are perpendicular to each other, and as such the dot product between them is supposed to be null.

(12) $$error=X\cdot Y=X^{T}Y$$

We create two new vectors to reduce the orthogonality error as:

(13) $$X_{ortho}=X-\frac{error}{2}Y$$

(14) $$Y_{ortho}=Y-\frac{error}{2}X$$

Considering that the magnitude of the column vectors are approximately equal to one, the dot product between the two new column vectors shows that the error is greatly reduced: the new error is now equal to $\left(1/4\right)error^{2}$.

The third orthogonal vector is created by taking the cross product between the two first:

(15) $$Z_{ortho}=X_{ortho}\times Y_{ortho}$$

The last step of the renormalization is used to ensure that the three column vectors have a magnitude equal to one. We apply a magnitude adjustment to the three orthogonal vectors as following:

$$X_{norm}=\frac{1}{2}\left(3-X_{ortho}\cdot X_{ortho}\right)X_{ortho}$$

$$Y_{norm}=\frac{1}{2}\left(3-Y_{ortho}\cdot Y_{ortho}\right)Y_{ortho}$$

(16) $$Z_{norm}=\frac{1}{2}\left(3-Z_{ortho}\cdot Z_{ortho}\right)Z_{ortho}$$

The renormalized rotation matrix is now formed by three new columns, where:

(17) $$R=\left(\begin{array}{ccc} X_{norm} & Y_{norm} & Z_{norm} \end{array}\right)$$

#### Drift correction

As stated above, the angular velocity given by the gyroscopes can contain bias and we need to add a correction to the angular velocity that is applied in the direction cosine matrix every time step:

(18) $$\omega =\omega_{gyro}+\omega_{correction}$$

The magnetometer and accelerometer values are used as a reference. The magnetometers are used as a reference to correct yaw and the accelerometers as a reference to correct pitch and roll. Each of the rotational drift correction vectors (yaw corrector eY and pitch-roll correctorePR) are fed to a proportional plus integral feedback.

The proportional correction is defined as:

(19) $$\;\omega_{P}=K_{P}^{yaw}eY+K_{P}^{pitchroll}ePR$$

The integral correction is defined as:

(20) $$\omega_{I}=\omega_{I}+K_{I}^{yaw}\;dt\;eY+K_{I}^{pitchroll}\;dt\;ePR$$

The controller gain $K_{p}$ and $K_{I}$ are respectively the gain proportional and the gain integral. The values chosen in our case are$\;K_{P}^{yaw}=1.2$,$\;K_{P}^{pitchroll}=0.02$,$\;K_{I}^{yaw}=0.001$ and $K_{I}^{pitchroll}=0.001$.

The total gyroscopic correction is then simply the addition of the two previous corrections:

(21) $$\omega_{correction}=\omega_{P}+\omega_{I}$$

#### Yaw Correction

The three axis magnetometer measures the magnetic field of its environment. The magnetometer was used to correct for drift in the measurement of yaw. If we consider the magnetometer mounted on the head in an environment with only the geomagnetic field as the magnetic source, we can write the two vectors representing the magnetic field measured by the sensor respectively in the head frame and the inertial frame as:

$$M_{H}=\left(\begin{array}{c} M_{x}\\ M_{y}\\ M_{z} \end{array}\right)$$

(22) $$\;M_{I}=\;\left(\begin{array}{c} B_{x}\\ 0\\ B_{z} \end{array}\right)$$

Note that the horizontal component of the geomagnetic field always points to the magnetic north pole aligned with the x axis of the inertial frame.

We can then write the relation between those two vectors and the rotation matrix:

(23) $$M_{H}=\;RM_{I}=R_{x}\left(\phi \right)R_{y}\left(\theta \right)R_{z}\left(\psi \right)M_{I}$$

For more convenience, we can write the equivalent equation thanks to the inverse of the rotation matrices:

(24) $$R_{y}^{T}\left(\theta \right)R_{x}^{T}\left(\phi \right)M_{H}=\;R_{z}\left(\psi \right)M_{I}$$

From the two first rows of the vectors (Equation 24), we can deduce:

(25) $$\tan\;\psi =\frac{\sin\;\phi M_{z}-\;cos\phi M_{y}}{\cos\;\theta M_{x}+\sin\;\theta \;\sin\;\phi M_{y}+\sin\;\theta \;\cos\;\phi M_{z}}$$

Thus, we can deduce the yaw value from pitch and roll and the magnetometer readings.

The correction of yaw is carried out as follows:

The unit vector of the head frame x axis is defined as ${\vec{e}}_{Hx}$. The unit vector of the head frame x axis as viewed from the inertial frame is represented by ${\vec{e}}_{Hx}^{I}$. Thus we have:

(26) $${\vec{e}}_{Hx}^{I}=R^{T}{\vec{e}}_{Hx}^{H}=R^{T}\left(\begin{array}{c} 1\\ 0\\ 0 \end{array}\right)=\left(\begin{array}{c} R_{11}\\ R_{12}\\ R_{13} \end{array}\right)$$

Next, we define the yaw correction in the inertial frame as the cross product between the projection of the unit vector of the head’s x axis on the inertial frame xy plane and the unit vector of the magnetometer’s yaw value in the inertial frame.

(27) $$eY_{I}=\left(\begin{array}{c} R_{11}\\ R_{12}\\ 0 \end{array}\right)\times \left(\begin{array}{c} \cos\;\psi_{m}\\ \sin\;\psi_{m}\\ 0 \end{array}\right)=\;\left(\begin{array}{c} 0\\ 0\\ R_{11}\;\sin\;\psi_{m}-R_{12}\;\cos\;\psi_{m} \end{array}\right)\;$$

Then we calculate the yaw correction in the head frame:

(28) $$eY_{H}=ReY_{I}=\left(\begin{array}{c} R_{13}\left(R_{11}\;\sin\;\psi_{m}-R_{12}\;\cos\;\psi_{m}\right)\\ R_{23}\left(R_{11}\;\sin\;\psi_{m}-R_{12}\;\cos\;\psi_{m}\right)\\ R_{33}\left(R_{11}\;\sin\;\psi_{m}-R_{12}\;\cos\;\psi_{m}\right) \end{array}\right)\;$$

The calculated value $eY_{H}$ is then used for drift correction, as in Equation 19 and 20.

#### Pitch-roll correction

The pitch and roll correction is carried out using the output from the accelerometers, which measure the difference between the linear acceleration of the sensor and the local gravity field.

We defined the accelerometer data from the head frame and from the inertial frame as:

(29) $$A_{H}=\left(\begin{array}{c} A_{Hx}\\ A_{Hy}\\ A_{Hz} \end{array}\right)$$

The pitch and roll correction is carried out by comparing the value for pure gravity from the accelerometers (which assume zero linear acceleration) with the unit vector of the z axis of the inertial frame, as viewed from the head frame $\left({\vec{e}}_{Iz}^{H}\right)$. The normalized accelerometer vector should be equal to the unit z axis vector of the inertial frame if both are viewed from the inertial frame.

$$ePR={\vec{e}}_{Iz}^{H}\times A_{H}=R{\vec{e}}_{Iz}^{I}\times A_{H}$$

(30) $$ePR=\;\left(\begin{array}{c} R_{13}\\ R_{23}\\ R_{33} \end{array}\right)\times \left(\begin{array}{c} A_{Hx}\\ A_{Hy}\\ A_{Hz} \end{array}\right)$$

The calculated value ePR is then used for drift correction, as in Equation 19 and 20.

#### Calibration of the inertial sensors

Prior to use the sensors require calibration. While the calibration values should not change drastically over time, they should be checked and recalibrated on a regular basis, especially if the local electromagnetic signals change over time (i.e., calibration values may be different depending on whether or not the sensor is used in conjunction with electrophysiological recordings).

Calibrations were carried out using open source Arduino software designed for the development of Attitude or Heading Reference Systems (AHRS; github.com/razor-AHRS). Accelerometers and gyroscopes were calibrated using the AHRS Arduino scripts.

Magnetometer calibrations were carried out separately using the Processing sketchbook (processing.org; software and instructions available at github.com/razor-AHRS). The magnetometer calibration is used to account for the presence of hard-iron and soft iron effects. Hard-iron calibration is considered to remove constant magnetic field affecting the sensor platform. Soft-iron calibration is required to eliminate the effects of electromagnetic fields.

#### Validation tests of inertial sensor system

The validity and reliability of the inertial sensor system for measuring head movements was tested in two ways: either with the sensor fixed in static position or undergoing rotations at a range of velocities.

To test for drift while in static regime the sensor was fixed to a base plate in the recording arena and recorded for 20 minutes while held in position. Measurements of the position in yaw, roll and pitch were made every 20ms. Drift in the system was tested by determining: (i) the shift in the sensor output between each measurement (jitter) (ii) the cumulative change in heading over the course of the twenty minute recording (cumulative drift). These measures test for the stability of the system in terms of bin-by-bin jitter and the presence of a directional bias in the jitter.

Tests of the validity of recordings during motion were carried out by comparing the sensor measurement with the expected motion elicited by a step-motor controlled rotation table. The step-motor system provides zero-acceleration/zero-deceleration (excluding the first and last steps) rotations around 360°. This was set at four different speeds (28°/s, 40°/s, 56°/s and 80°/s) considered to be representative of a range of potential movement velocities of the head, and was set to rotate either clockwise or counter-clockwise.

The inertial sensor was fixed to the rotation table and recordings were carried out nine times at each direction and velocity. The expected angular displacement between each 50Hz measurement (28°/s = 0.56°, 40°/s = 0.8, 56°/s = 1.12° and 80°/s = 1.6°) was then compared with the computed displacements between each temporal bin from the sensor output. The measurement of error was then transformed to give a measurement error per degree for each temporal bin. These were then compared using a two-way 4x2 factorial ANOVA.

#### Electrodes and surgery

Mice were implanted with moveable 17μm-diamteer platinum-iridium (H-ML insulated) microelectrodes (California Fine Wire, US), configured as four tetrodes and carried by 16 channel microdrives (Axona, St. Albans, UK). Tetrodes were platinum electroplated to an impedence of 100-250 kOhm using a Kohlraush/Gelatin (9:1, 0.5% gelatin) solution. Electrodes were implanted just ventrally to the intermediate layers of the superior colliculus at co-ordinates 3.8-4.2 mm posterior from Bregma, 1.25 mm lateral of the midline and 1.3-1.5 mm ventral to the brain surface. All mice were given at least one week to recover before recording commenced.

#### Apparatus and recording environment

Single-units were recorded as mice foraged a square (50 × 50 cm) Perspex arena for droplets of 30% diluted soya milk. Recording sessions consisted of four five-minute foraging trials, with the first and last occurring in light conditions and the second and third occurring in complete darkness. The recording arena itself was situated within a Faraday cage containing stable polarizing cues. Light trials were recorded with one door of the Faraday cage open, while the arena was completely enclosed during dark trials. During dark trials all other sources of light within the experimental room such as computer screens were switched off or covered.

#### Recording procedures

Single-unit recording was carried out using a multi-channel DacqUSB recording system (Axona, St Albans, UK). In order to record units, animals were connected to a pre-amplifier via a lightweight cable attached to the microdrive by a headstage that modified the signal with AC-coupled, unity gain operational amplifiers. The signal was amplified ∼12-20000 times and bandpass filtered between 500Hz and 7 kHz. Recording thresholds were set to ∼70% above baseline activity levels, and data from spikes above the threshold from all channels were collected across a period spanning 200μs preceding and 800μs following the peak amplitude of a spike. The activity of channels from any given tetrode was referenced against the activity of a single channel from another tetrode, so as to increase the signal to noise ratio. Tetrodes were advanced ventrally into the brain by 50-100μm after each recording session.

#### Integration of inertial sensor and single-unit recordings

The inertial sensor was attached to the headstage on the head of the mice using Mill-Max connectors. The signal from the sensor was passed through a lightweight cable via one Arduino for processing the signal and computing the DCM algorithm (described above) and a second for controlling synchronization with the DacqUSB single unit recording system. The control Arduino was connected to the DacqUSB system using the system’s Digital I/O port. A custom built BASIC script was written in DacqUSB to synchronize the start of single-unit recording with the key-press initiation of inertial sensor recording (controlled using the Processing software sketchbook; processing.org)

#### Spike sorting

The electrophysiological data were spike sorted using Tint cluster cutting software (Axona, St Albans, UK. Cluster cutting was carried out by hand as clusters were generally well separated. Clusters were included in analysis if they exhibited over 100 spikes during the light trial recording sessions and did not belong to clusters identified in previous recording trials.

#### Histology

Animals were sacrificed once tetrodes were estimated to have passed beyond the superior colliculus. Mice were anaesthetized under 3% isofluorane before receiving intraperitoneal injections of 0.1ml of Pentabarbital Sodium (Euthetal). Mice were then transcardially perfused using phosphate buffered solution followed by the fixative solution formalin (∼4% formaldehyde). Brains were stored in the fixative and then 20% sucrose solution for 24 hours in order to cryoprotect the tissue. Brains were subsequently frozen at −20°C before 30μm coronal sections were cut. The sections were then Nissl-stained using thionine solution. A light microscope fitted with a digital camera was used to determine tetrode position. The images of electrode tracks were then referenced against images taken from the Mouse Brain Atlas [29] in order to estimate electrode position within the superior colliculus. Images were converted to greyscale for presentation purposes.

### Quantification and Statistical Analysis

All inferential statistical analyses were performed in Python or Graphpad Prism. All other analyses were written in bespoke scripts in Python. All statistics are reported alongside n values as mean ± SEM in the Results section of the manuscript and in the figure legends. Repeated-measures ANOVA underwent Greenhouse-Geisser corrections and pairwise t tests were Bonferroni corrected for multiple comparisons.

#### Determining head motion events

Head motion events for each Eulerian component were defined as events in which the angular head velocity remained in a constant direction for at least five temporal bins (a total of 100ms) at a speed of over 0.5 of a degree per bin (25°/s). This definition was further refined by searching backward from the onset of the initially defined motion to the last temporal bin at which direction was the same as the defined motion; this was now defined as the onset of motion. A similar process was also carried out to define the offset of motion, the last temporal bin from the initially defined offset of motion to have the same direction as the defined motion was considered as the final offset of motion. From these values we retrieved the total extent of motion (the summation of the angular head velocity for a motion event) and the duration of motion for each motion event. This process was carried out separately for each Eulerian component. This definition of movement angles excludes any movements of 2.5° or under so as to prevent the inclusion of data that might come about due to small jitters of the sensor.

#### Individual component analysis

Frequency histograms were created for the head displacements for each animal, taken from the light trial recordings. The computed head displacements were grouped into 36 ten degree bins and normalized based on the maximum sampling frequency for the creation of the frequency histograms. Gaussian curves were fit to the resulting distributions and the peak, mean and sigma of the fit were retrieved from fitted model. Repeated-measures ANOVA were used to compare standard deviations of the fit curves.

#### Conjunctive motion analysis

For the defined motion events in any given Eulerian component we calculated the head displacement in each of the other components, as described above. Any motion events that began in more than one component simultaneously were only counted once. Sampled head motions were then grouped into a 36 × 36 matrix of bins for each pair of Eulerian components (yaw x pitch, yaw x roll and pitch x roll) for each animal. The frequency of samples in each bin were then transformed into a log10 scale and plotted as a heatmap, with warmer colors representing higher frequencies and white areas representing non-sampled displacements.

Linear regressions were carried out on the non-transformed data for each animal in order to determine whether there was a systematic bias in the direction and extent of conjunctive motion. Repeated-measures ANOVA were used to compare the R^2^ and coefficient values between motion types and light conditions.

#### Quaternion representation of head motion

The quaternions used for our analysis are unit four-vectors of the form:

(31) $$q\left(\alpha ,\;\bar{n}\right)=q_{0}+q=\cos\frac{\alpha }{2}+\sin\frac{\alpha }{2}\bar{n}$$

where α is the angle performed around the rotation axis and $\bar{n}$ is the unit three-vector indicating the direction of the rotation axis.

The three components of the vector part ***q*** are *q*_*T*_, *q*_*V*_, *q*_*H*_. A rotation around the x axis of the coordinate system is represented by the term *q*_*T*_, defined also as the torsional component; the vertical component *q*_*V*_ is a rotation around the y axis; *q*_*H*_ is defined as a horizontal rotation around the z axis. The conceptualization of quaternions follows from Euler’s theorem, which states that if a point in a rigid body moving in a three-dimensional space remains in a fixed position, then the movement of the object can be seen as a rotation around an axis that runs through the fixed point. The theorem can be applied to the relative movement between two 3-dimensional coordinate systems having the same origin (in our case, the inertial reference frame and the local head reference frame). This displacement can be described as a single rotation α around an axis passing through the origin and α results to be the combination of three rotations (Euler angles; where *ψ* is yaw, *θ* is pitch and ϕ is roll) around each of the axes of the initial coordinate system. The relationship between Euler angles and quaternions is expressed by the following formulae:

$$q_{0}=cos\frac{\phi }{2}cos\frac{\theta }{2}cos\frac{\psi }{2}+sin\frac{\phi }{2}sin\frac{\theta }{2}sin\frac{\psi }{2}$$

$$q_{T}=sin\frac{\phi }{2}cos\frac{\theta }{2}cos\frac{\psi }{2}-cos\frac{\phi }{2}sin\frac{\theta }{2}sin\frac{\psi }{2}$$

$$q_{V}=cos\frac{\phi }{2}sin\frac{\theta }{2}cos\frac{\psi }{2}+sin\frac{\phi }{2}cos\frac{\theta }{2}sin\frac{\psi }{2}$$

(32) $$q_{H}=cos\frac{\phi }{2}cos\frac{\theta }{2}sin\frac{\psi }{2}-sin\frac{\phi }{2}sin\frac{\theta }{2}cos\frac{\psi }{2}$$

Note that the relationships above take into account the fact that the z axis of our coordinate system is rotated 180 degrees (as defined by the reference frame of the inertial sensor) compared to the one of previous studies, which used two search coils in three magnetic fields to compute head movements [28, 42, 43, 44, 45].

#### Quaternion visualization

Once the quaternion components were estimated, results were plotted. Quaternions are four-dimensional vectors, which represent a hypersphere, called three sphere (or *S*^3^). This means that their representation should occur in a 4-dimensional space *R*^4^. However, since our quaternions are unit vectors, the component $q_{0}$ can always be written as a function of the other three components:

(33) $$q_{0}=\pm \sqrt{1-q\cdot q}=\pm \sqrt{1-q_{T}^{2}-q_{V}^{2}-q_{H}^{2}}$$

Therefore, it is possible to plot quaternions in a 3D space by representing only their vectorial part, as $q_{0}$ is redundant. The quaternions’ space can be drawn as two 3D spheres (a northern and a southern hemisphere), where the external surface represents those points for which ‖q‖ = 1 and $q_{0}$ = 0, and the origin coincides with ***q*** = ($q_{T}$,$\;q_{V}$,$\;q_{H}$) = (0,0,0) (and, consequently, |*q*_0_| = 1). In the northern hemisphere, the origin assumes the value *q*_0_ = 1, whereas in the southern hemisphere, its value is *q*_0_ = −1.

In our representation, only the northern hemisphere is considered, that is equivalent to choosing only the positive roots of *q*_0_. Quaternion data were plotted in the 3-dimensional space as vectors that indicate the direction of the rotation axis and have a length equivalent to the sine of half of the angle of the rotation performed (α).

#### Listing’s plane rotation

The quaternion values obtained from Equation (32) are expressed with respect to our reference position; that is when the head of the mouse is aligned with the x axis of the inertial frame (pointing forward, toward North). However, the reference position chosen does not necessarily correspond to the primary position *r*; that is when the mouse’s head orientation is perpendicular to the Listing’s plane (if Listing’s law holds). Indeed, the rotation axes associated with movements from and to the reference position lie on a plane, called the displacement plane (*DP*_ℎ_). This plane is not perpendicular to the reference position unless the reference position corresponds to the primary position [42]. To facilitate the interpretation of the data, the quaternions’ values are therefore rotated so that reference position corresponds to primary position, with Listing’s plane coinciding with the plane y-z of our reference coordinate system (where *q*_*T*_ = 0). The transformation is performed by following a method applied previously [42].

A corollary of Listing’s law is that, for any head position *h*, if $\bar{v}h$ is the unit vector perpendicular to the displacement plane *DP*_*h*_ associated with the position *h*, $\bar{v}h$ is also the bisecting line of the angle between the head direction vector ${\bar{d}}_{h}$ and the primary head direction vector ${\bar{d}}_{P}$. From this, it derives that the head primary position calculated relative to the position ℎ is:

(34) $$p={\bar{v}}_{h}\cdot {\bar{d}}_{h}-{\bar{v}}_{h}\times {\bar{d}}_{h}$$

Therefore, in order to rotate our data and refer them to primary position, it is first necessary to find that primary position for which Equation (34) is valid. We start assuming as reference position *r* that orientation of the mouse’s head for which the head points toward North (x axis of our inertial frame). First, we calculate the quaternions relative to *r*, and subsequently we fit the data with a plane, expressed by:

(35) $$q_{T}=a_{1}+a_{2}q_{V}+a_{3}q_{H}$$

If the parameter *a*_1_ is different from zero, then the reference position chosen does not respect the corollary of Listing’s law. However, that position for which the corresponding quaternion is $h=\left(\sqrt{1-a_{1}^{2},}a_{1},0,0\right)$ does lie in plane (5). Therefore, the position *h* can be taken as the new reference position. This was done by right-multiplying our quaternion results by the inverse of the quaternion representing the new reference position (ℎ^−1^).

The results of the product are again fitted by a plane $\left(q_{Th}=a_{1h}+a_{2h}q_{V}+a_{3h}q_{H}\right)$, in order to calculate the new displacement plane (*DP*_*h*_). However, if ℎ^−1^ is of a small entity, the new plane can be considered to be the same as the plane obtained by Equation (35). In our case, the average difference between the planes *q*_*T*_ and *q*_*Th*_ was 1.27° (maximum, 2.73°), and as such we used the new displacement plane (*DP*_*h*_) for subsequent calculations. First we calculated its normal vector:

(36) $$V_{h}=\frac{\left(1,a_{2h},a_{3h}\right)}{\left|1,a_{2h},a_{3h}\right|}=\left(\frac{1}{\sqrt{1+{\left(a_{2h}\right)}^{2}+{\left(a_{3h}\right)}^{2}}};\;\frac{a_{2h}}{\sqrt{1+{\left(a_{2h}\right)}^{2}+{\left(a_{3h}\right)}^{2}}};\frac{a_{3h}}{\sqrt{1+{\left(a_{2h}\right)}^{2}+{\left(a_{3h}\right)}^{2}}}\right)=\left(V_{1},V_{2},V_{3}\right)$$

Note that the normal vector components here have a different sign compared to the ones reported previously [42]. Indeed, our reference system is rotated by 180° compared to the one adopted previously. Once the normal vector to *DP*_*h*_ is determined, results are rotated to primary position by left-multiplying them by the inverse of the quaternion of primary position (*P*^−1^) relative to *h*:

(37) $$p=V_{h}\cdot i-V_{h}\times i=\left(V_{1,}0,V_{3,\;}-V_{2}\right)$$

where ***i*** is a unit vector aligned with the x axis of the reference position (***i*** = (1,0,0)). The alignment of reference and primary position is meaningful to better estimate the torsional component of the movement. Indeed, if the data are computed compared to the reference position, it is difficult to define quantitatively the amount of torsion, being the x axis non-orthogonal to the displacement plane.

#### Surface fitting

To verify whether the results respected Donder’s law, they were fitted with a first-order surface (a plane) and a second-order surface using the least-squares minimization method. For head in space movements, 1000 points for each mouse were considered to compute the surface. The surfaces are of the type:

(38) $$q_{T}=a_{1}+a_{2}q_{V}+a_{3}q_{H}$$

and:

(39) $$q_{T}=a_{1}+a_{2}q_{V}+a_{3}q_{H}+a_{4}q_{V}^{2}+a_{5}q_{V}q_{H}+a_{6}q_{H}^{2}$$

The term *a*_5_ in Equation (39) gives an estimate of the twist observed in the generated surface, and it is called twist-score. The parameter was found to assume negative values in previous studies [28, 44, 46] and this was interpreted as indicative of a Fick gimbal-like distribution of the data. However, we found that the surfaces which best fitted our data did not present a clear pattern of *a*_5_ values across mice (see Figure S2). The data were also fitted also with Fick surface, of the form:

(40) $$q_{T}=s\left(\frac{q_{V}q_{H}}{q_{0}}\right)$$

where the parameter *S* is the so-called gimbal score. If *S* = –1, then the surface represents a perfect Fick gimbal, whereas if *S* = 1, head movements follow a Helmholtz gimbal (a vertical axis is nested within a fixed horizontal axis). If *s* is equal to zero the surface coincides with a Listing’s plane. To estimate how well the data were fitted by the surfaces, we computed the torsional standard deviation (Tsd), which measures the standard deviation of the scatters between the actual *q*_*T*_ component of our data and the *q*_*T*_ of the points lying on the surface. The estimated Tsd values were compared to the ones previously encountered in studies on primates.

#### Determination of motion tuning

The motion tuning of superior colliculus neurons was determined by carrying out burst triggered averages of head displacements. In order to do this, we first defined bursting events, then aligned corresponding head displacements to these events and compared the computed average displacement vectors with those drawn from a random distribution. Cells were only considered to be tuned to a given component if the motion vector of the cell for that component was above threshold (described below) and in the same direction in both light trials.

#### Burst analysis

Bursting events were defined as spiking epochs in which a cell fired three or more spikes, with a maximum of 50 ms between spikes and a minimum duration of bursting event of 20 ms. Only cells exhibiting five or more bursting events in each recording session were tested for motion tuning.

#### Burst triggered averages

For the burst triggered average of motion the angular head velocity for the 25 (500ms) temporal bins preceding and 50 bins (one second) following the onset of bursting were computed for each Eulerian component. The direction of the head at the onset of each burst onset was normalized to zero for each Eulerian component. The calculated angular head velocities (AHV) were cumulatively summated for each temporal bin to produce a head displacement for the 500ms preceding and one second following the onset of bursting. This was repeated for each bursting epoch. The mean and s.e.m. of burst related head displacements were then calculated for each temporal bin to illustrate the tuning of neurons.

Displacement vectors were calculated as the difference between the minimum and maximum of the computed displacement (between burst onset and 500ms following burst onset) for each bursting event, and the mean displacement vector for each Eulerian component was computed from each neuron’s computed displacement vectors for the given component. The direction of the displacement vector was defined according to the temporal order of the minimum and maximum values of the computed displacement (i.e., if the minimum preceded the maximum value, the displacement was deemed to be positive).

#### Generation of shuffled datasets

Neurons were defined as being motion tuned if their mean displacement vectors fell outside of 95% of data drawn from a shuffled distribution. These shuffled distributions were computed for each cell separately.

For each cell, the burst-onset times were temporally shifted by 20-150 s (selected from a random distribution) in a wrap-around manner. This works to shift the relationship between the bursting times and the recorded heading directions of the animals while maintaining the temporal relationship between bursting events. Once these data were shifted, burst triggered analyses (as described above) were carried out to determine the mean displacement angle of the temporally-shifted data. This process was repeated 1000 times so as to produce a random distribution of mean displacement vectors.

Neurons were considered to be motioned tuned if their mean-displacement fell either under the 2.5% or above the 97.5% points of the randomly generated distribution of mean displacement vectors in the same direction in both light trials.

#### Effect of bursting epochs on tuning

For each motion-tuned neuron we calculated the number of bursts occurring in light trials and dark trials.

The mean displacement angles in the first light trials were then calculated for spikes occurring inside and outside of bursting epochs. This analysis was done in the same way as described for burst triggered averages, but rather than aligning movement onsets to the onset of bursting this alignment was done to spike times. This was done for within-burst spikes and non-burst spikes separately, and the resultant mean displacement angles were compared using paired t tests. The percentage of spikes occurring within bursts were calculated as well as the firing rate of bursting events and the mean interspike intervals of spikes occurring within bursts.

#### Comparisons of tuning extent

To compare the within-cell variability of mean displacement angles with the between-cell variability we first calculated the absolute difference in mean displacement angle for each modulated cell between the first and second light trial. We next calculated the difference of the absolute displacement angle between the first light trial each modulated cell and that of all other cells with modulation to same Eulerian component. The mean difference between cells was then calculated and paired t tests were used to compare the within-cell differences with the between-cell differences.

#### Testing effect of motion sampling on tuning

Gaussian curves were fit to the frequency histograms of spatial sampling for the first light trial of each motion-tuned neuron. The sigma of the fit curves were used as a measure of motion sampling range. Linear regression was used to determine relationship between motion sampling in the first light trial and each cell’s displacement tuning in the same trial. This was done separately for each Eulerian component.

#### Temporal characteristics of motion-tuning

The temporal characteristics of motion tuning were assessed by first separating movement epochs (definition described above) into the two directions of motion for each Eulerian component and sorted in the order of movement duration. The temporal bins (relative to motion onset) of the onset bursting events were then found for the 500ms prior to and following the onset of motion. The total number of bursts within this temporal range was then calculated for each temporal bin (20ms bins) and was then used to calculated the z-score of bursting events for each bin.

We next compared the average z score of bursting for each motion-tuned cell across four 80ms long temporal windows, with temporal bins beginning −160ms to −100ms, −80ms to −20ms, 0ms to 60ms and 80ms to140ms). For cells with tuning to more than one Eulerian component the mean z-score of bursting for each component was averaged to prevent over sampling of individual cells. Repeated-measures one-way ANOVA were used, followed by Bonferroni correct pairwise t tests.

#### Effect of rate and burst duration on tuning

The rate (spikes/second) and duration of bursting (in seconds) were also calculated. The motion vector associated with each busting event was then tested for correlation with the rate and duration of bursting using linear regression. The reported coefficient values for the rate correlation are shown in degrees for each Hz, while the coefficients for the duration correlation are shown in degrees for each 10ms.

#### Analysis of angular head velocity

Angular head velocity (AHV) was derived from the differences between heading angle for each temporal bin in each Eulerian axis separately. AHV per temporal bin was then multiplied by 50 (the sampling rate of the inertial sensor) to provide AHV values in degrees per second. This data was then smoothed over time using a rectangular window with a bin width of five temporal bins (100ms). The number of spikes for each motion-tuned cell was calculated for each temporal bin. The AHV data was then binned into fifty 20°/second bins with a range of −500°/s to 500°/s and the total number of spikes occurring in each AHV bin was calculated. Firing rate was determined as the number of spikes per second, and accounted for the amount of time spent in each AHV bin.

For comparisons between models of AHV firing rate modulation the data were first normalized by the bin with the maximum firing rate. The python package LMfit was used to fit either a constant or skewed Gaussian distribution to the normalized firing rate data. Least-squares fitting was used to fit the models to the data within the given parameters. The constant model predicted no modulation of firing rate by AHV and thus had only one parameter, the y-intercept of the model. The skewed Gaussian model was fit using four parameters: the center (μ), amplitude (*A*), sigma (σ) and gamma (γ) values, according to the equation:

$$f\left(x;A,\mu ,\sigma ,\gamma \right)=\frac{A}{\text{σ}\sqrt{2\text{π}}}e\left[{\left(x-\mu \right)}^{2}/2{\text{σ}}^{2}\right]\left\{1+erf\left[\frac{\gamma \left(x-\mu \right)}{\text{σ}\surd 2}\right]\right\}$$

Where erf() is the error function and x is the array of AHV bin centers

Initial parameters were defined with a center and amplitude of 0. The minimum and maximum of the center of the distributions were set to −500° and 500° degrees. The initial value of the sigma parameter was 200, with minimum and maximum values of 100° and 500° respectively. The initial gamma parameter for skewness was set at 0, with a maximum value of ± 5. The fit of the models were compared using Bayesian information criterion, and cells were considered tuned to AHV if the BIC scores for the skewed Gaussian model less than the constant model by ten or more and if the center of the fit Gaussian was in direction of the tuned displacement vector revealed using the burst triggered analysis.

The BIC is defined as:

$$\text{BIC}=\ln\left(n\right)k-2\text{ln}\left(\hat{L}\right)$$

where $\hat{\text{L}}$ is the maximized value of the likelihood function of the model, *n* is the number of AHV bins, and *k* is the number of parameters estimated by the model. In our case, these are one for the constant model and four for the skewed Gaussian model.

#### Generation of allocentric tuning curves

The head-direction tuning curves of neurons were computed by binning the firing frequency based upon heading direction into bins of 10° each for azimuth and 5° each for bank and elevation. 36 bins were created for the full 360° of sampling in the azimuth (yaw) component, while 20 bins ranging from −45° to 45° degrees were created for elevation (pitch) and bank (roll) components. The firing rates of neurons were then calculated by dividing the total number of spikes in each directional bin by the dwell time (in seconds) in each bin. As the full circularity of the azimuth angle was readily sampled by mice, circular statistical methods were used to define the tuning of cells to azimuth [47]. For elevation and bank, we adopted methods used previously in the determination of tuning for these components [4].

#### Rayleigh vector analysis of azimuth heading

Rayleigh vector lengths were calculated as:

(41) $$r=\sqrt{x^{2}+y^{2}}$$

Where x and y are the rectangular co-ordinates of the mean tuning angle. Rayleigh vector values range from 0 to 1, with values closer to 1 indicating strong directional modulation and values close to 0 indicating that the firing rate is uniform with respect to heading angle. Rayleigh vector scores were compared to scores drawn from a shuffled distribution. Shuffling procedures were as described above for the burst triggered average analyses. Only cells with a Rayleigh vector score above 95% of the shuffled distribution in both light trials and with a difference of preferred tuning direction (bin of maximum firing rate) between light trials of under 30° were considered to have modulation by azimuth.

#### Analysis of allocentric pitch and roll heading

The maximum firing rate for pitch and roll were calculated separately for each cell. The tuning width of cells was calculated as 25% of the maximum firing rate; where the maximum firing rate is the difference between the firing rate at the directional bin with the highest firing rate and the rate at the bin with the lowest firing rate. This was done to discount background firing rates of the cell from analyses, as has been done previously [4]. The percentage of bins which exceeded this threshold was then calculated. A cell was considered to be directionally tuned if it’s tuning width was smaller than 5% of the tuning widths drawn from a shuffled distribution (calculated as above) and if the difference in preferred firing directions between light trials was under 30°.

#### Anticipatory effects of azimuth modulated cells

Spikes of azimuth modulated cells were temporally shifted in 20ms bins between −100ms and 100ms from their original spike times. Rayleigh vector scores were calculated (as above) for each temporal shift providing scores of azimuth tuning at different time points relative to heading angle. An increase in Rayleigh vector scores for positively shifted time points would indicate anticipatory firing of azimuth modulated cells, while an increase for negatively shifted time points would indicate delayed firing of azimuth tuned cells [33]. One-way repeated-measures ANOVA were used to compare Rayleigh vector scores for these different time points.

### Data and Software Availability

The data and code that support the findings of this study are available from the Lead Contact upon reasonable request.

## Supplemental Information

Document S1. Figures S1–S6 and Tables S1 and S2

Document S2. Article plus Supplemental Information
