# Supplementary material for: Three-Dimensional Representation of Motor Space in the Mouse Superior Colliculus
Source: Curr Biol. 2018 Jun 4;28(11):1744–1755.e12. doi: 10.1016/j.cub.2018.04.021 (PMC5988568; doi:10.1016/j.cub.2018.04.021)
Supplement: Document S1. Figures S1–S6 and Tables S1 and S2 [file mmc1.pdf]

Current Biology, Volume *28*

Supplemental Information

Three-Dimensional Representation of Motor Space in the Mouse Superior Colliculus

Jonathan J. Wilson, Nicolas Alexandre, Caterina Trentin, and Marco Tripodi

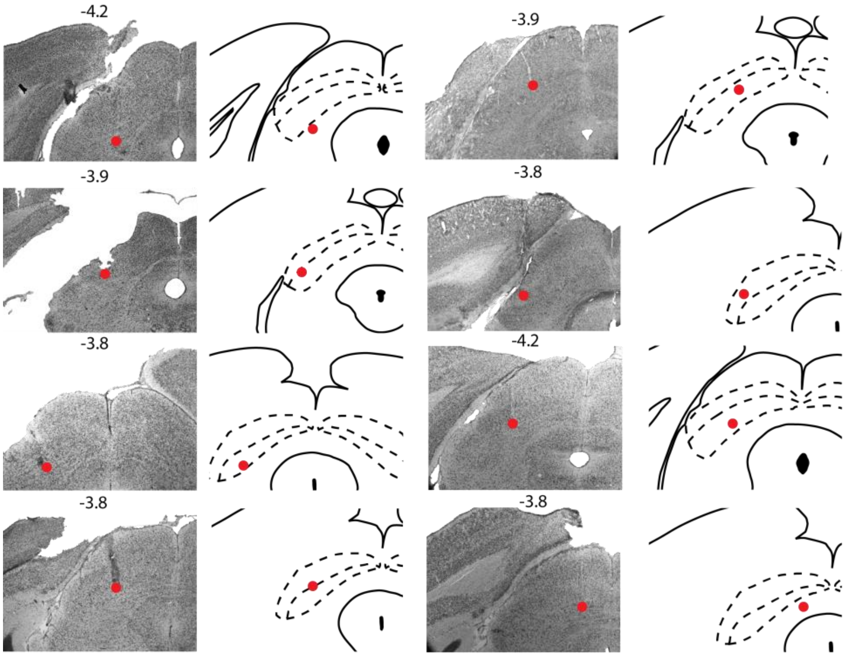

Figure S1. Photomicrographs of electrode location. Related to Figure 3 Thionine stained brain sections showing the locations of electrodes within the SC (left) and the estimated location of electrodes highlighted on a brain atlas (modified from Paxinos & Franklin [S1]) for the eight recorded mice. Numbers indicate the estimated posterior distance from Bregma in millimetres.

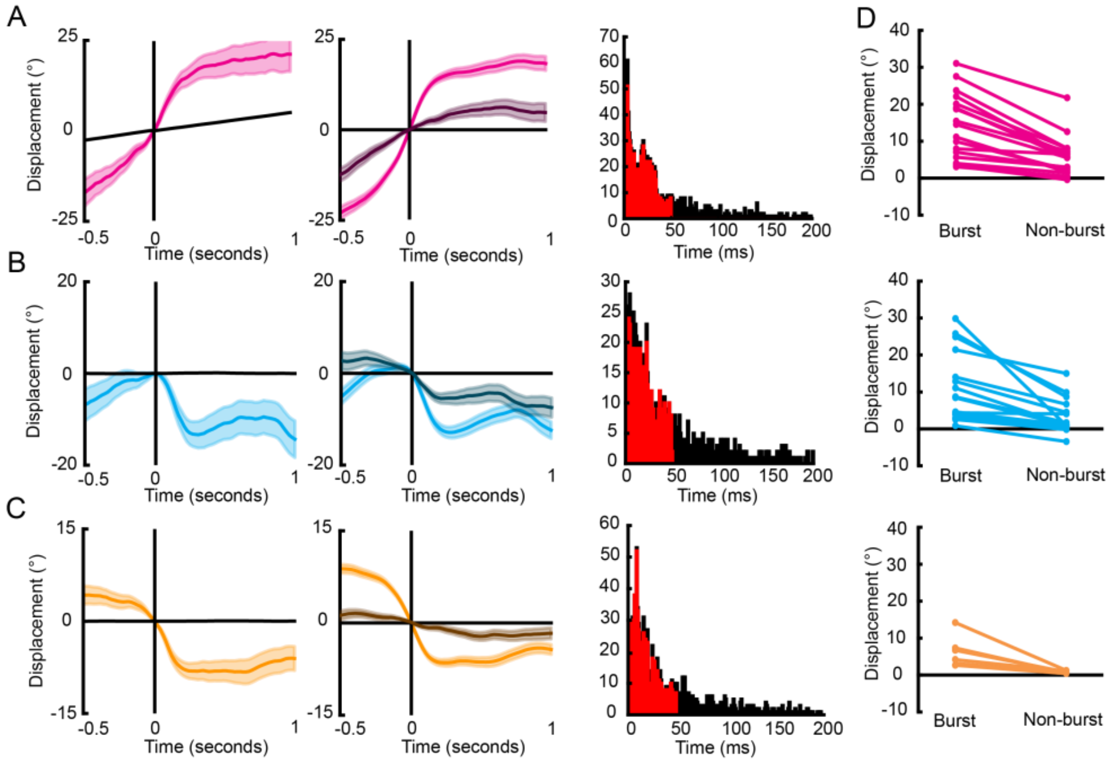

Figure S2. Spikes within bursting epochs contribute more to angular displacement. Related to Figure 3. (A-C) Left – burst triggered average plots with head displacements aligned to the onset of bursting (vertical black line). Centre – spike triggered average plots shown for spikes falling inside bursts (light shade) and for spikes falling outside of bursts (darker shades), for a yaw –tuned (A), pitch-tuned (B) and roll-tuned (C) cell. Note the greater average tuning for spikes falling within bursting epochs. Right – interspike-interval histograms for the same three cells showing the inter-spike intervals for spikes falling inside bursts (red) and spikes falling outside of bursts (black). (D) Mean displacement angle for yaw tuned (top), pitch-tuned (middle) and roll-tuned neurons for spikes inside and outside of bursting epochs - note the loss of tuning for non-burst spikes.

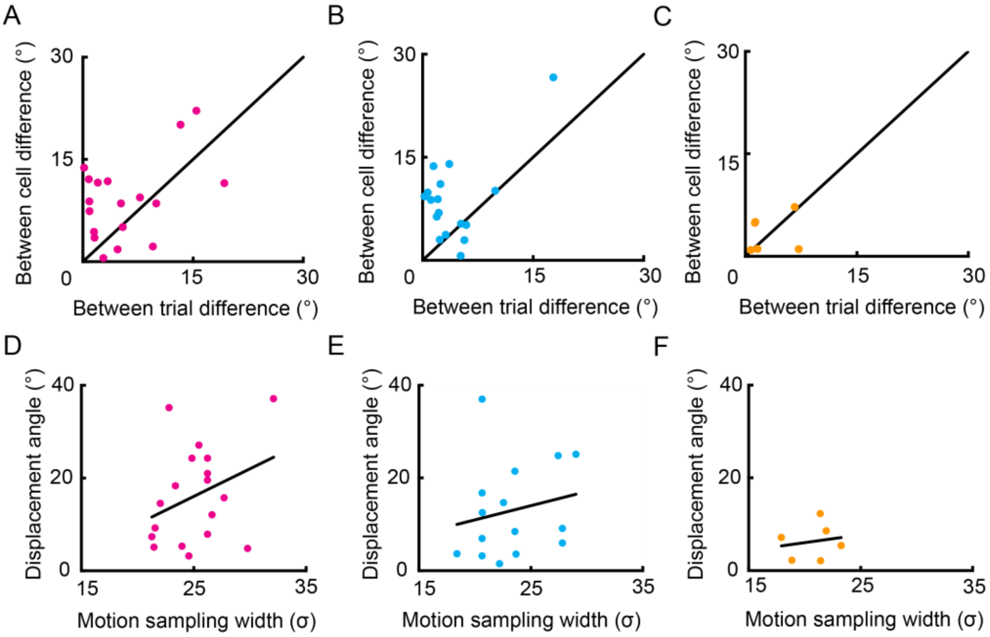

Figure S3. Stable preferred displacement angles of motion-tuned neurons. Related to Figure 3. (A-C) Scatter plots showing the differences in preferred displacement angles of each motion-tuned cell between the two light trials compared with their average difference to other motion-tuned cells shown for yaw (A), pitch (B) and roll (C). Points falling above the diagonal black line indicate greater between cell variability than between trial variability. (D-F) Scatter plots showing the absolute displacement angles of motion tuned cells for yaw (D), pitch (E) and roll (F) compared to the sigma width of Gaussian curves fitted to the motion sampling data from the recorded trials. Black line shows the trend line resulting from linear regression. There was no effect of the range of motion sampling on the displacement angles of motion tuned cells.

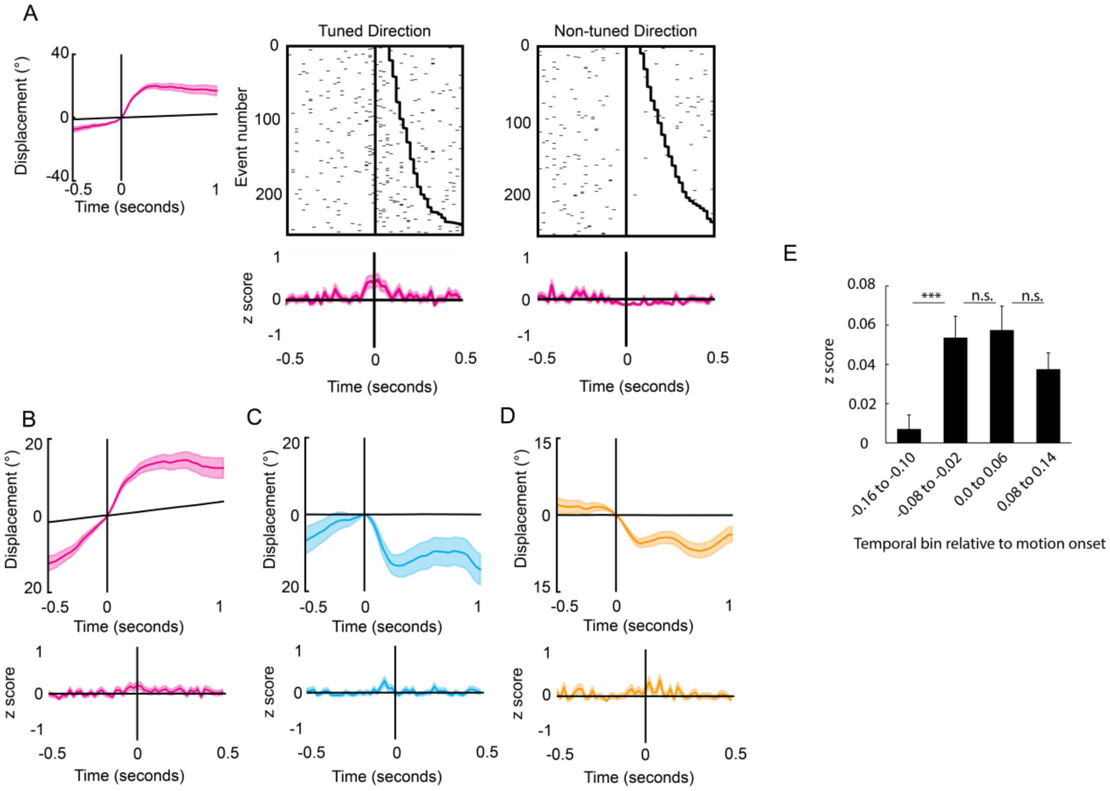

Figure S4. Temporal dynamics of tuning. Related to Figure 3. (A) Example burst triggered analysis (BTA) of a yaw tuned cell (left) and associated plot showing the onset of bursting (black marks), depicting the z score of bursting for the cell 0.5 seconds prior to and 0.5 seconds following the onset of motion angular head displacements in the cell's tuned direction (left) and non-tuned direction (right). Vertical black line shows onset of motion and curved black line shows the offset of motion. Mean ± SEM. of z-scores are shown beneath for each temporal bin. Note that for the tuned direction there is an increase in bursting prior to the onset of motion (before the vertical axis). (B-D) BTAs (top) and plots showing average z-score of bursting event (below; shown for the tuned direction only) of a cell's with tuning to yaw(B), pitch (C) or roll (D). (E) Bar charts showing the average z-scores of bursting events for four time points (two preceding and two following onset of motion). Note the increase in bursting in the time points prior to the onset of motion.

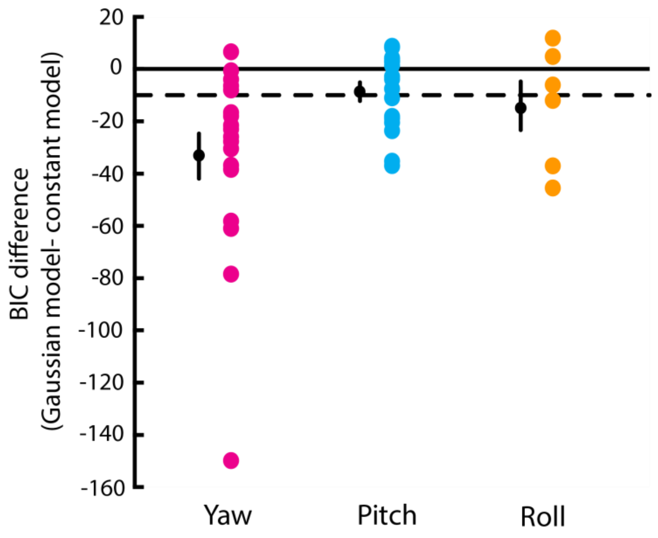

Figure S5. Model comparisons for angular velocity tuning. Related to figure 4. BIC score differences between the Gaussian model and constant model of velocity tuning, shown for neurons with yaw, pitch or roll tuning. Motor tuned neurons with Gaussian model BIC scores of at least 10 fewer (black dashed line) than the constant model were considered to have firing rate tuning to angular head velocity. Mean ± SEM shown in black.

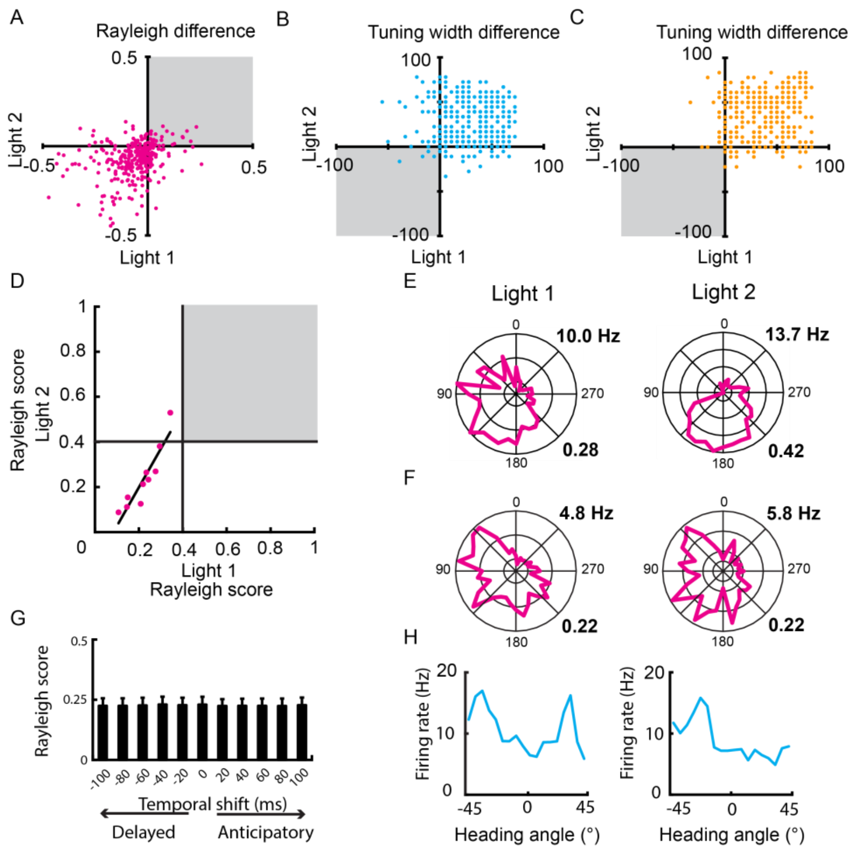

Figure S6. Allocentric tuning for all recorded SC neurons. Related to Figure 6. (A-C) Scatter plots showing the difference in tuning between the recorded data and the shuffled distributions for each recorded neuron in each light trial. (A) For azimuthal tuning, rayleigh vector scores were compared. Only neurons with higher Rayleigh vector scores in both light trials than the shuffled distributions (grey box) and a consistent preferred firing direction (within 30°) were considered to be modulated by azimuthal heading. (B-C) For elevation (B) and bank (C) tuning, only cells with lower tuning widths in both light trials (grey box) and a consistent preferred firing direction (within 30°) were considered to be modulated. (D) Of the azimuth modulated neurons no cells exhibited Rayleigh scores above 0.4 in both light trials (grey box) indicating only a low level modulation by azimuthal heading. (E-F) Polar plots of two azimuth modulated neurons for both light trials. Firing rates (top right) and Rayleigh scores (bottom right) are shown in bold for each plot. (G) Analysis of anticipatory time intervals for azimuth modulated neurons revealed no increase in Rayleigh vectors for anticipated heading or past heading. (H) Example of one cell exhibiting modulation of firing rate by elevation heading for light trial 1 (left) and light trial 2 (right).

Table S1

Mouse	Tsd^{1st} (º)	Tsd^{2nd} (º)	Tsd^{Fick}(º)
**M1**	23.61	19.56	22.24
**M2**	32.41	27.73	31.09
**M3**	21.12	18.39	17.83
**M4**	23.56	21.21	23.21
**M5**	25.00	24.04	24.37
**M6**	26.07	26.04	25.31
**M7**	24.28	24.26	23.63
**M8**	23.48	23.46	23.59
**M9**	20.73	20.58	20.74

Table S1. Measures of fit of quaternion data. Related to figure 2. Values of Torsional standard deviations (Tsd) from the light trials of each mouse, shown for each type of fit (1st order, 2nd order or Fick surfaces) to the quaternion data.

Table S2

**Previous studies**	**Tsd^{1st} (º) (mean ± SD)**	**Tsd^{2nd} (º) (mean ± SD)**
Straumann et al. (1991) [S2]	1.3±0.5	-
Glenn and Vilis (1992) [S3]	-	2.56±0.53
Radau et al. (1994) [S4]	-	4.75±2.58
Tweed et al. (1995) [S5]	-	Mean range: 2.4-2.6
Crawford et al. (1999) [S6]	-	3.1
Klier et al. (2003) [S7]	-	3.09±0.46 M1<br>6.5±0.69 M2

Table S2. Values of torsional standard deviations (Tsd) from previous studies. Related to figure 2. Note the low values in comparison with those shown in Table S1.

Supplemental References

S1. Paxinos, G., and Franklin, K.B.J. (2001). The mouse brain in stereotaxic coordinates (second edition), (Academic Press).

S2. Straumann, D., Haslwanter, T., Hepp-Reymond, M.C., and Hepp, K. (1991). Listing's law for eye, head and arm movements and their synergistic control. Exp Brain Res *86*, 209-215.

S3. Glenn, B., and Vilis, T. (1992). Violations of Listing's law after large eye and head gaze shifts. Journal of Neurophysiology *68*, 309-318.

S4. Radau, P., Tweed, D., and Vilis, T. (1994). Three-dimensional eye, head, and chest orientations after large gaze shifts and the underlying neural strategies. J Neurophysiol *72*, 2840-2852.

S5. Tweed, D., Glenn, B., and Vilis, T. (1995). Eye-head coordination during large gaze shifts. Journal of Neurophysiology *73*, 766-779.

S6. Crawford, J.D., Ceylan, M.Z., Klier, E.M., and Guitton, D. (1999). Three-dimensional eye-head coordination during gaze saccades in the primate. J Neurophysiol *81*, 1760-1782.

S7. Klier, E.M., Wang, H., and Crawford, J.D. (2003). Three-dimensional eye-head coordination is implemented downstream from the superior colliculus. J Neurophysiol *89*, 2839-2853.
